# Maternal demographic and antenatal factors, low birth weight and preterm birth: findings from the mother and child in the environment (MACE) birth cohort, Durban, South Africa

**DOI:** 10.1186/s12884-020-03328-6

**Published:** 2020-10-16

**Authors:** Prakash M. Jeena, Kareshma Asharam, Aweke A. Mitku, Pragalathan Naidoo, Rajen N. Naidoo

**Affiliations:** 1 Discipline of Paediatrics and Child Health, Nelson R Mandela School of Medicine, University of KwaZulu-Natal, Durban, South Africa; 2 Discipline of Occupational and Environmental Health, School of Nursing and Public Health, Howard College Campus, University of KwaZulu-Natal, Room 321, George Campbell Building, Durban, 4041 South Africa; 3 Discipline of Medical Biochemistry and Chemical Pathology, Howard College Campus, University of KwaZulu-Natal, Durban, South Africa

**Keywords:** Age, Cigarette smoking, Caesarean delivery, Preterm birth, Low birthweight

## Abstract

**Background:**

Low birthweight (LBW) and preterm birth (PB) remain the leading cause of morbidity and mortality in neonates worldwide. The aim of this study was to identify maternal demographic and antenatal factors associated with PB and LBW among low socio-economic communities.

**Methods:**

Pregnant women (*n* = 1099) were recruited in the first trimester into the Mother and Child in the Environment (MACE) birth cohort in Durban, South Africa. Maternal factors such as demographic information, health status, residential area, occupational, personal and environmental smoking and biomass fuel use were obtained through standardised interviews, while clinical status was obtained in each trimester and antenatal information on HIV status and treatment, syphilis and conditions such as pregnancy induced hypertension, diabetes etc. was extracted from the antenatal assessments. Key outcomes of interest were preterm birth and low birthweight. The latter data was obtained from the clinical assessments performed by midwives at delivery. Logistic regression models identified factors associated with PB and LBW.

**Results:**

Of the 760 live births, 16.4 and 13.5% were preterm and LBW, respectively. Mothers who delivered by caesarean section had an increased odds of having LBW babies (Adjusted odds ratio (AOR): 1.7; 95% CI: 1.1–2.7) and PB (AOR: 1.7, 95% CI: 1.1–2.7) versus normal vaginal deliveries. Mothers > 30 years (AOR: 1.8, 95% CI: 1.1–2.9) and current smokers (AOR: 2.7, 95% CI: 1.3–5.8) had an increased odds of having PB babies. Compared to younger mothers and non-smokers respectively. An effect of PB and LBW was seen among mothers with high BMI (25.0–29.9 kg/m^2^) (PB: AOR: 0.5, 95% CI: 0.3–0.9 and LBW: AOR: 0.5, 0.5, CI: 0.3–0.8), and obese BMI (> 30 kg/m^2^) (PB: AOR: 0.5, 95% CI: 0.3–0.9 and LBW: AOR: 0.4, CI: 0.2–0.7). Maternal HIV (PB AOR: 1.4 and LBW AOR: 1.2) and history of sexually transmitted infections (PB AOR: 2.7 and LBW AOR: 4.2) were not statistically significant.

**Conclusion:**

Maternal age, cigarette smoking and caesarean delivery were associated with LBW and PB. Findings highlight the need of maternal health interventions to improve new-born health outcomes.

## Background

Globally, around 30 million low birthweight (LBW) babies and 15 million preterm births (PB) are born annually. This accounts for 23.4% and 10–11% of all births, respectively [1, 2]. In sub-Saharan Africa, adverse pregnancy outcomes including spontaneous abortion, PB (defined as birth prior to 37 weeks of gestation), LBW (defined as birthweight < 2500 g), intrauterine growth retardation (IUGR), small for gestational age (SGA) babies, stillbirths and babies with congenital anomalies are triggered by a broad spectrum of obstetric risk factors such as hypertension, gestational diabetes, tuberculosis and sexually transmitted infections (HIV/AIDS and syphilis) [3–6]. In addition, factors such as poverty, maternal malnutrition, unhealthy living conditions (ambient air pollution and poor sanitary conditions) and tobacco smoke exposure can also play a role in the prevalence of adverse birth outcomes [3–6].

In South Africa, the neonatal mortality rate in 2017 was reported as 12 per 1000 live births [5]. Hypertension, sepsis, haemorrhage and sexually transmitted infections are the main contributing factors for maternal deaths, while among births greater than 500 g, PB (22.9%), unexplained intrauterine deaths (22.8%), intrapartum asphyxia (13.4%) and antepartum haemorrhage (10.6%) were the leading causes of neonatal deaths [7]. In the province of KwaZulu-Natal (KZN), approximately 180 maternal deaths for every 10,000 live births, and 70 neonatal deaths for every 1000 live births are seen [8].

South African women experience multiple burden of diseases, including those driven by high prevalence of HIV and tuberculosis [6, 7], poor socio-economic status [9] and lack of access to healthcare [10]. The prevalence of tuberculosis in KZN is estimated to be 1094 cases/100000 population and 12.9% of general population in KZN are HIV infected [11].

Despite the evidence supporting socio-economic factors such as maternal education and household income have been associated with low birthweight outcomes, specific household and environmental factors (housing type, biomass exposure and environmental tobacco smoke) have been reported in limited studies in low socioeconomic communities, with a combined high prevalence of established factors such as HIV positive status, poor obstetric history and poor nutritional status. Being able to adjust for these important risk factors, within a sample of low socioeconomic status participants, particularly from the sub-Saharan sub-continent is necessary to understand these risks.

Investigating maternal risk factors that contribute to PB and LBW are compromised in cross-sectional studies, primarily through recall bias. A cohort, in which females, recruited early in the first trimester, and risk factors assessed throughout the gestational period, provide distinct advantages. Our objective in this study was to describe maternal demographic and antenatal factors that are possibly associated with adverse birth outcomes, PB and LBW, in a longitudinal Mother and Child in the Environment (MACE) birth cohort in Durban, South Africa.

## Methods

### Selection of communities, study population and study sample

Pregnant women attending the public sector ante-natal clinics in Durban and surrounding areas (Lamontville, Merebank, Bluff, Austerville, Wentworth, KwaMashu, Newlands East and Newlands West), and who met the inclusion criteria were invited into the cohort. The objective of the MACE study was to determine the risk of environmental pollutant exposure commencing in utero on long term respiratory health of children to 6 years of age and specific outcomes such as asthma.

The selection criteria for subjects to enter this study were as follows:

- The recruited participants had to be residing in the geographical area within which the clinic is located, and had to live in this area for the full duration of the pregnancy and follow-up period of up to 5–6 years. The children had to be resident in the communities for the duration of follow-up (to monitor the health of the child from birth up to 6 years of age, and to keep track of their health by gathering information from their Road to Health charts).
- Pregnant females had to preferably be less than 20 weeks of gestational age on entry although those presenting before the onset of the 3rd trimester were not excluded.

Women who were asthmatic, hypertensive, diabetic or tested positive for human immunodeficiency virus (HIV), syphilis and tuberculosis during their routine ante-natal testing were included. Participating females with complications of pregnancy such as preeclampsia, placenta previa, were also included. Females with multiple pregnancies were excluded from the study.

### Sample selection

**Fig. 1:**
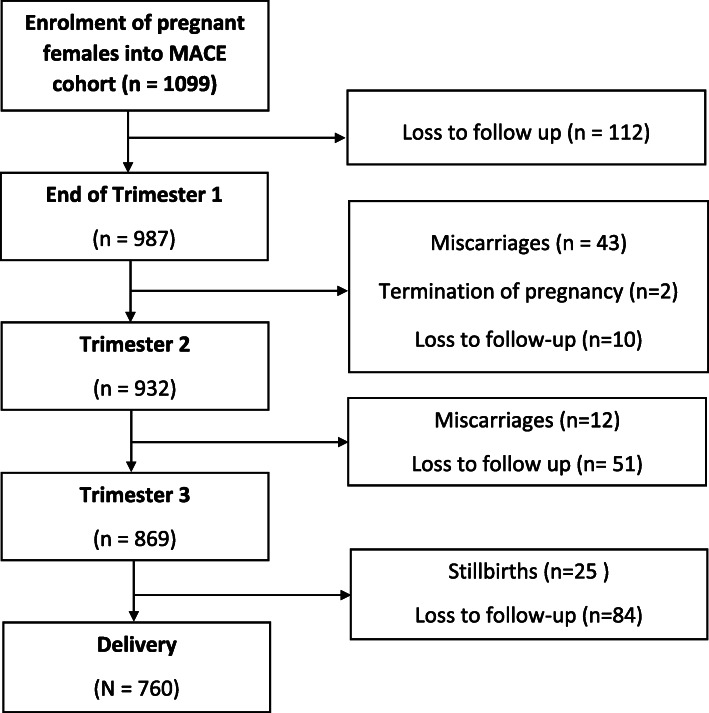
Cohort profile illustrating hierarchical number of participating women used in the study from the MACE birth cohort

We report on the first 1099 recruited pregnant women who had progressed to delivery, experienced a stillbirth or miscarriage, or exited the cohort. The cohort profile (Fig. 1) provides details of the changes from the point of recruitment in April 2013 through to March 2018. The cohort size at each trimesters of pregnancy were 987 (1st trimester), 932 (2nd trimester), 869 (3rd trimester), and 760 (at delivery). Relocation of participants outside the study area and choosing to use clinics closer to their new homes was the single largest reason for the loss to follow-up (*n* = 257). Participants (*n* = 82) who experienced miscarriages and stillbirths or terminating their pregnancy were subsequently removed from the cohort.

### Maternal interviews and antenatal clinical assessments

Trained interviewers conducted standardised validated face-to-face interviews with all mothers at enrolment, second and third trimesters, as well as monthly telephonic follow-up interviews. The questionnaires included demographic information, antenatal history, place of birth and residential history, occupational history, maternal smoking, exposure to passive cigarette smoke, occupational and environmental exposures, use of biomass fuels, dietary history, pre-existing medical conditions, and past medical and obstetric history. Maternal demographic information was based on responses to questions about marital status (married/living together/single/divorced/widowed); maternal education (highest grade of school completed/post-high school education/degree), maternal income (six categories of annual income ranging from no income to US$7200 or greater) and maternal employment (student/apprentice/at home/unemployed/disabled/ choice of seven key sectors of work).

Data was captured at the time of the interview using a mobile telephone system, automatically uploading data onto the study database using wireless technology, Mobile Researcher®.

All questionnaires were made available in English and isiZulu (an indigenous South African language). The isiZulu versions were translated from English and then back translated by a second translator to assure that the instruments are truly equivalent.

A clinical assessment of all enrolled subjects were undertaken in the 3rd trimester of pregnancy. Maternal weight was assessed at enrolment and at delivery. Obesity was evaluated just prior to delivery. In addition, information on routine assessments performed as part of their antenatal follow-up by the public health service was extracted from their records. These included their HIV status and if positive, use of antiretroviral medication, tests for syphilis and diabetes.

### Postnatal data collection

Time of birth and delivery type data were collected by the fieldworkers or extracted from the labour records. Anthropometric measurements and gestational aging were done by the hospital nursery staff using World Health Organisation (WHO) standards. The midwife undertook the measurement of birthweight for each child independently while early ultrasound and post-natal scoring measured gestational age. The person undertaking these measurements was blinded to the study outcomes. This data were used to classify cases into those that were PB (< 37 completed weeks of pregnancy) and those with LBW (< 2500 g). Gestational age was estimated by a combination of clinical date of last menstrual period, early antenatal ultrasound and the Ballard scoring system postnatally. A combination of prematurity and weight status per individual child was not done.

### Statistical analysis

PB and LBW were the dependent variables of interest. A variety of independent variables were explored through interviews using the standardised instruments. These variables included: socio-demographic variables (marital status; maternal education, maternal income, maternal employment); maternal general, reproductive and obstetric health; residential variables (residential area, housing type; energy type); behaviour and exposure in the current pregnancy (alcohol and smoking history, environmental tobacco smoke exposure, biomass fuel use). In addition, variables with known association with the outcomes of interest were included in our analyses. These included maternal age, HIV positive status, antiretroviral treatment and weight gain during pregnancy.

Standard exploratory data techniques were employed, and descriptive statistics, with frequencies and ranges for categorical, mean and standard deviation (SD) for continuous variables were used to summarize participants’ characteristics.

The variables listed above, which, in the bivariate analyses showed a *p*-value < 0.25 (allowing for joint effects) were included in the multivariate logistic regression models. We ran separate models for each of the dependent variables of interest, PB and LBW respectively. We additionally included those variables which were clinically important into the models, such as maternal age. We report the crude and adjusted odds ratios (AORs) from the logistic regression models with their 95% Confidence Interval (CI). A *p*-value less than 0.05 was considered to be statistically significant. The IBM SPSS statistical software package (version 25.0) was used for all statistical analyses.

### Ethics approval and consent to participate

The study was approved by the Biomedical Research Ethics Committee (BREC) of the University of KwaZulu-Natal (UKZN) (BF263/12). All the participants in this study gave written informed consent, participated voluntarily, received no financial incentives, and had the right to withdraw at any stage.

## Results

### Baseline demographic data of the cohort

The majority of mothers were single (79.0%); 16.7% had received tertiary education, over 84.0% did not work, 44.0% earned a yearly gross income of less than R2 000, with most living in low-income housing communities (67.2%) and informal settlements (14.7%) (Table 1). Of those who worked, almost all (98.2%) had low to middle income jobs (Table 1).

**Table 1:** Demographic data for pregnant mothers in the MACE birth cohort (*n* = 1099^a^)

Characteristics	
Mean age in years (SD)	26.1 (5.9)
Teenager (15–19 years) (%)	87 (11.5)
Prime of fertility (20-30 years) (%)	487 (64.4)
Middle aged (> 30 years) (%)	182 (24.1)
Marital Status	
Married (%)	154 (14.0)
Living together (%)	77 (7.0)
Single (%)	868 (79.0)
Mean number of family members per household (SD)	3.1 (1.6)
Housing Type	
Detached house, Semidetached (%)	739 (67.2)
Flat, Terraced flat, Apartment building (%)	198 (18.0)
Informal (%)	162 (14.7)
Mean number of rooms per household (SD)	3.3 (1.9)
Ventilation (*n* = 950)	
Natural (%)	886 (93.3)
Other (%)	64 (6.6)
Cooking Energy Source (*n* = 950)	
Electricity (%)	922 (97.6)
Biomass fuels (%)	18 (2.0)
Paraffin (%)	12 (1.3)
Other (%)	6 (0.6)
Mother working (*n* = 165, 16.1%)	
General assistant (%)	56 (33.9)
Clerical/administrative (%)	19 (11.5)
Machine operator/Supervisor/manager (%)	13 (7.9)
Other (%)	76 (46.1)
Mothers Education	
Never attended or only up to grade 1–7 (%)	28 (2.5)
Some High School education (%)	269 (24.5)
Matric (high school graduate) (%)	619 56.3)
College/ Technikon/ University (%)	183 (16.7)
Mothers Annual Income:	
< R2000 (%)	484 (44.0)
R2001- R10000 (%)	195 (17.7)
R10 001 - R30 000 (%)	238 (21.7)
> R30 000 (%)	121 (11.0)
Refused to answer (%)	61 (5.6)
Mean Gestational week at enrolment (SD)	11.6 (3.5)
Mean Gravida (SD)	2.5 (0.9)
Mean Parity (SD)	0.7 (0.9)
Primigravida (%)	486 (44.2
Planned pregnancy (%)	439 (39.0)
Use of contraceptive in the past year (%)	545 (49.6)
Pregnant on contraception (%)	242 (22.0)
Sexual relationship with baby’s father (%)	
For some months (%)	176 (16.0)
For some Years (%)	923 (84.0)
Sexual intercourse during the 4 weeks before pregnancy	
Refused to answer (%)	67 (6.1)
> 3 times a week (%)	445 (40.4)
1–2 times a week (%)	332 (30.2)
< 1 time a week (%)	255 (23.2)
Outcomes of previous pregnancies (*n* = 965)^b^ in 613 mothers	
Live birth (%)	801 (83.0)
Spontaneous Abortion (%)	109 (11.3)
Termination of pregnancy (%)	15 (1.6)
Ectopic pregnancy (%)	20 (2.1)
Still birth (Macerated and fresh) (%)	4 (0.4)
Postnatal Death (%)	21 (2.2)
Patients with bad obstetric history (*n* = 613) (%)	148 (24.1)

*SD* Standard deviation

^a^ unless otherwise stated

^b^613 mothers reported having a previous pregnancy

The mean gravida and parity scores were 2.5 (SD: 0.9) and 0.7 (SD: 0.9) respectively, with 44.2% of mothers having their first pregnancy. (Table 1). Within the sample, 11.5 and 24.1% were teenage and middle aged pregnancies. While 39.0% of pregnancies were planned, 22.3% occurred on contraception usage. Over 70% of the cohort were sexually active for a minimum of once per week. Of the 613 participants with a previous obstetric history (*n* = 965 previous pregnancies), 24.0% had bad obstetric outcomes; with the majority (11.3%) having spontaneous abortions (Table 1).

Almost half (45.3%) of the pregnant mums lived with people that were cigarette smokers and approximately a third (31.2%) of them were exposed to environmental tobacco smoke (ETS) (Table 2). A small subset of the cohort were ever-smokers (7.0%) and only 3.6% actively smoked during the pregnancy. A small proportion (7.4%) were ever consumers of alcohol and only 2.7% actively drank during the pregnancy (Table 2).

**Table 2:** Maternal behavioural and pregnancy factors among the pregnant females in the MACE birth cohort

	N (%)
Alcohol and Smoking factors (*n* = 869)	
Presence of cigarette smokers in home	413 (45.3)
Passive Smoking	271 (31.2)
Mother Ever Smoked	61 (7.0)
Current smoker	31 (3.6)
Ever Alcohol use	64 (7.4)
Current Alcohol use	23 (2.6)
Prevalence of co-morbid diseases (*n* = 880)	
HIV^a^	261 (30.9)
Syphilis ^b^	90 (15)
TB	16 (1.8)
PIH	15 (1.7)
Other STI’s	9 (1.0)
Diabetes	5 (0.6)
Mean weight gain (SD)	6.3 (6.1)
Maternal BMI at trimester I	
Low BMI (< 18.5 kg/m^2^)	33 (4.4)
Normal BMI (18.5–24.9 kg/m^2^)	252 (33.7)
High BMI (25–29.9 kg/m^2^)	216 (28.9)
Obese	247 (33.0)
Delivery type	
NVD	491 (64.6)
Caesarean	269 (35.4)

*HIV* Human immunodeficiency virus; *TB* Tuberculosis; *PIH* Pregnancy induced hypertension; *STI* Sexually transmitted infections; *NVD* Normal vaginal delivery

^a^HIV (*n* = 828)

^b^Syphilis (*n* = 693)

A third of the study population were infected with HIV (30.9%) and all were receiving antiretroviral therapy (ART), while 15% tested positive for syphilis. The prevalence of hypertension, gestational diabetes, tuberculosis and asthma were extremely low (Table 2). The mean weight gain for mothers across the first to the third trimester of pregnancy was 6.3 (SD: 6.1 kg) (Table 2).

### Birth outcomes

From the enrolled cohort of 1099 cases, a total of 760 (70.8%) live births resulted. As expected, the incidence of miscarriages were higher in the first trimester compared to the second trimester of pregnancy (4.0% versus 1.1% respectively). There were 2.3% of mothers who experienced stillbirths during the third trimester. The occurrence of neonatal and infants deaths were low at 0.7 and 1.4% respectively (Table 3). Over 16.4% of mothers delivered PB and 13.5% of babies that were delivered were LBW (< 2500 g); 0.6% were < 1500 g. (Table 3).

**Table 3:** Birth outcomes in the current pregnancy in the MACE birth cohort (*n* = 1074)

Birth Outcomes	N (%)
First trimester^a^	
Miscarriage	43 (4.0)
Termination of pregnancy	2 (0.2)
Second trimester^b^	
Miscarriage	12 (1.1)
Third trimester^c^	
Still birth	25 (2.3)
Live births	760 (70.8)
Death	22 (2.1)
Neonatal Death (< 28 days)	7 (0.7)
Infant Death (28 days-1 year)	15 (1.4)
**Gestational Age (*n*** **= 760)**	
Preterm birth (**≤37 weeks**)	125 (16.4)
Normal birth (38–42 weeks)	632 (83.3)
Postdate birth (> 42 weeks)	3 (0.4)
**Birth Weight (*n*** **= 760)**	
VVLBW (< 1000 g)	2 (0.3)
ELBW (1001 g–1500 g)	2 (0.3)
LBW (**1501*−*** 2499 g)	98 (12.9)
Normal birth weight (2500 g–4000 g)	620 (81.5)
High birth weight (> 4000 g)	38 (5.0)

Notes: ^a^ Loss to follow up in the first trimester = 112 (10.5).

^b^ Loss to follow up in the second trimester = 55 (5.2).

^c^ Loss to follow up in the third trimester = 63 (5.9).

*VVLBW* Very, very low birth weight; *ELBW* Extreme low birth weight; *LBW* Low birth weight; *LGA* Large for gestational age

### Factors associated with PB and LBW

From the bivariate analyses, variables of household risk (location, type or energy type) and socio-demographic variables (marital status; maternal education, maternal income, maternal employment) did not reach statistical significance, and were therefore not included in the logistic regression models.

Mothers aged > 30 years (AOR: 1.8, 95% CI: 1.1–2.9), current smokers (AOR: 2.7, 95% CI: 1.3–5.8) and caesarean deliveries (AOR: 1.7, 95% CI: 1.1–2.7) had statistically significant increased odds ratios for PB. Although not significant, mothers that were teenagers (15–19 years) (AOR: 1.2, 95% CI: 0.6–2.3), were HIV positive (AOR: 1.4, 95% CI: 1.0–2.2), and with a history of tuberculosis (AOR: 2.0, 95% CI: 0.6–6.6) and sexually transmitted infections (AOR: 2.7, 95% CI: 0.6–12.2) also had increased odds ratios for PB babies. Conversely, overweight mothers (BMI: 25.0–29.9 kg/m^2^) (AOR: 0.5, 95% CI: 0.3–0.9), obese mothers (BMI: > 30 kg/m^2^) (AOR: 0.5, 95% CI: 0.3–0.9) and mothers with a low BMI (< 18.5 kg/m^2^) (AOR: 0.9, 95% CI: 0.4–2.4) did not present with increased odds of having PB babies (Table 4).

**Table 4:** Factors associated with Preterm Birth and Low Birth Weight (*n* = 760)

	Crude Odds Ratio	*p*-value	Adjusted Odds Ratio (95% CI)	*p*-value
**Preterm Birth**				
Maternal age				
Prime fertility age (20–30 years)	1		1	
Teenager (15–19 years)	1.2	0.5	1.2 (0.6–2.3)	0.6
Middle aged (> 30 years)	1.7	0.02	1.8 (1.1–2.9)	0.01
Maternal BMI at trimester 1				
Normal BMI (18.5–24.9 kg/m^2^)	1		1	
Low BMI (< 18.5 kg/m^2^)	1.4	0.4	0.9 (0.4–2.4)	0.9
High BMI (25–29.9 kg/m^2^)	0.6	0.03	0.5 (0.3–0.9)	0.02
Obese (> 30 kg/m^2^)	0.6	0.03	0.5 (0.3–0.9)	0.01
Past history of tuberculosis	3.5	0.02	2.0 (0.6–6.6)	0.3
Past history of sexually transmitted infections	2.6	0.2	2.7 (0.6–12.2)	0.2
Syphilis	0.3	0.02	0.4 (0.1–0.9)	0.04
Hypertension				
Normotensive				
Chronic	0.7	0.8	0.8 (0.09–6.7)	0.8
PIH	1.1	0.9	0.5 (0.06–4.2)	0.5
HIV positive	1.4	0.1	1.4 (1.0–2.2)	0.1
Current Smoker	3.22	0.001	2.7 (1.3–5.8)	0.01
Delivery Type				
Normal delivery	1		1	
Caesarian section	1.54	0.03	1.7 (1.1–2.7)	0.01
**Low Birth Weight**				
Weight gain				
Normal	1		1	
High (> 6.3 kg)^a^	0.74	0.2	0.6 (0.4–1.03)	0.07
Maternal age				
Prime fertility age (20–30 years)	1		1	
Teenager (15–19 years)	1.7	0.1	1.6 (0.9–3.1)	0.1
Middle aged (> 30 years)	1.4	0.1	1.7 (1.0–2.9)	0.07
Maternal BMI at trimester 1				
Normal (18.5–24.9)	1		1	
Low BMI (< 18.5 kg/m^2^)	1.7	0.2	1.3 (0.5–3.3)	0.5
High BMI (25–29.9 kg/m^2^)	0.5	0.01	0.5 (0.3–0.8)	0.01
Obese (> 30 kg/m^2^)	0.5	0.02	0.4 (0.2–0.7)	0.002
Past history of sexually transmitted infections	3.3	0.09	4.2 (1.0–18.3)	0.06
Syphilis	0.7	0.4	1.0 (0.6–1.7)	0.9
Hypertension				
Normotensive	1		1	
Chronic	Not available		Not available	
PIH	1.4	0.6	1.2 (0.1–10.6)	0.9
HIV Positive	1.2	0.5	1.2 (0.8–2.03)	0.4
Smoking				
Non-smoker	1		1	
Current-smoker	2.4	0.03	1.9 (0.8–4.3)	0.1
Delivery Type				
Normal delivery	1		1	
Caesarian section	1.4	0.1	1.7 (1.1–2.7)	0.03

^a^Mean weight gain across the first and third trimester for mothers during this pregnancy

- LBW (Yes = 1, No = 0), with “YES” when LBW < 2500 g

*CI* Confidence interval; *BMI* Body mass index; *Ref* Reference; *PIH* Pregnancy induced hypertension; *HIV* Human immunodeficiency virus; *NVD* Normal vaginal delivery

Mothers that had caesarean deliveries had an increased odds ratio for LBW babies (AOR: 1.7, 95% CI: 1.1–2.7), when compared to those with normal vaginal deliveries. Although not significant, pregnant mothers who were teenagers (AOR: 1.6, 95% CI: 0.9–3.1) and middle age (AOR: 1.7, 95% CI: 1.0–2.9), current smokers (AOR: 1.9, 95% CI: 0.8–4.3), low BMI (AOR: 1.3, 95% CI: 0.5–3.3), having a history of sexually transmitted infections (AOR: 4.2, CI: 1.0–18.3,), being HIV positive (AOR: 1.2, 95% CI: 0.8–2.0), or having pregnancy induced hypertension (AOR: 1.2, 95% CI: 0.1–10.6) also had increased odds of LBW babies. Conversely, overweight (AOR: 0.5, CI: 0.3–0.8) and obese mothers (AOR: 0.4, CI: 0.2–0.7) and having syphilis did not appear to influence the outcome. (Table 4).

## Discussion

This South African birth cohort study in Durban, found a strong association of maternal smoking during pregnancy, middle aged pregnancies, caesarean section deliveries and low BMI with preterm births. An equally interesting finding was that of a protective effect against both PB and LBW of a high BMI among pregnant mothers. The association of maternal smoking and PB was against the background of a low prevalence of current smoking.

These findings are replicated in other studies. A previous South African study showed that maternal ethnicity, height, BMI, socioeconomic status, and antenatal tobacco and alcohol use were significant modifiable predictors of birthweight [12]. Maternal tobacco use throughout pregnancy was associated with LBW [13], PB [14], and SGA births [13–15]. In most African countries, maternal knowledge on the harmful effects of tobacco use in the manifestation of adverse perinatal outcomes are extremely limited [16]. In our study, a strong association between mothers that were active smokers and PB outcomes was observed. Active smokers were also at increased odds of delivering LBW babies compared to non-smokers, although this association was not significant).

Increasing maternal age is also an independent risk factor for PB and LBW delivery. Studies amongst Americans of different race and ethnic groups, have shown that middle age mothers (> 30 years) were most at risk of delivering LBW babies [17, 18]. Teenagers (15–19 years) also had a significantly increased risk of delivering LBW babies as compared those at prime of fertility (25–29 years) while teenage and middle age expecting mothers were at a significantly higher risk of delivery PB babies compared to those at prime of fertility [19]. In our study, an association between middle age mothers and PB outcomes was observed, and although not significant, teenage and middle age mothers were also at risk of delivering LBW babies. In other studies adverse birth outcomes was most prevalent amongst middle age primigravidas with poor socioeconomic status, tobacco smoke exposure, stress and non-communicable diseases [20].

Although caesarean section emerged as an explanatory factor for both PB and LBW, it is more likely that these children are at greater risk for adverse birth outcomes during labour and thus emergency delivery of baby was necessary. As compared to normal vaginal delivery, caesarean section is considered more rapid and highly recommended when delivering PB and LBW babies as it helps reduce the risk of stillbirths and neonatal deaths [21].

Maternal nutrition and gestational weight gain have been considered to be important risk factors for delivering PB (overall, induced and spontaneous) and variable birth weight babies [22–24]. Maternal overweight and obesity did not have an adverse effect for LBW, but not for PB, in developing countries [25]. A meta-analyses of cohort studies have significantly associated maternal underweight with PB and LBW [26]. Other studies found that maternal underweight and poor nutritional status to be risk factors for PB and LBW, while obese pregnant women have a low prevalence of PB outcomes [27–29]. In our study, maternal overweight and obesity were not associated with PB and LBW. Our findings highlight the importance of proper maternal nutrition and appropriate weight gain during pregnancy to minimise the risk of adverse birth outcomes.

Even though, majority of mothers in our cohort were from poor socioeconomic backgrounds, our study failed to reproduce the findings from other studies where a significantly higher prevalence of LBW and PB were seen in women that were single and unmarried, and with minimal education and low income [30, 31]. Single, unemployed South African women who have experienced previous miscarriages are at high risk of having unplanned pregnancies [32, 33]. In our cohort, there was a high rate of unplanned pregnancies with 22.3% of women on contraceptives when they fell pregnant. This could imply contraceptive failure or non- adherence as described by other studies [34, 35].

The prevalence of HIV/AIDS and tuberculosis among pregnant South African women have rapidly increased over the past decades. HIV infected women are at a higher risk of delivering PB and LBW babies [36] and experiencing spontaneous abortion and stillbirth [37] and having early infant deaths [38] as compared to HIV uninfected women. Women who start ART before conception as compared to those who start after, are also at a higher risk of delivering LBW, preterm or very preterm babies [39, 40], experiencing spontaneous abortion, stillbirth, elective terminations and ectopic pregnancies [41], and experiencing unintended pregnancies due to contraceptive failures [42]. Similar adverse birth outcomes have been reported in mothers who had tuberculosis [43, 44]. Although no significant associations were observed in our cohort, maternal HIV infection and history of tuberculosis showed trend towards having PB and LBW outcomes in our study.

The prevalence of other sexually transmitted infections in pregnant South African women is also increasing amongst those < 25 years of age, unmarried and non-cohabiting women [45]. Higher incidence risks of sexually transmitted infections (Chlamydia trachomatis, Neisseria gonorrhoea, Trichomonas vaginalis and syphilis) was found in women who attended urban ante-natal clinics and who experienced perinatal death in their previous pregnancies and these cases have been associated with adverse birth outcomes, especially stillbirths [46, 47]. In Tanzania, mothers with high-titer active syphilis had a very high prevalence of stillbirths and were at a high risk of delivering LBW and PB babies as compared to mothers with other serological stages of syphilis [48]. In unscreened women a high prevalence of stillbirths, PB and other adverse birth outcomes have been attributable to maternal syphilis [49].

In our study, a trend between maternal history of sexually transmitted infections and PB/ LBW outcomes was observed. In contrast to previous reports, we found no effect of maternal syphilis infection on PB and LBW outcomes. Although the serological staging for syphilis was not determined, we speculate that mothers may have had low-titre infections, which could explain our findings.

### Strengths and limitations

A concern in our study was the high dropout rate of pregnant women from enrolment to delivery mainly due to relocation. However, this was likely to be random, and thus not expected to have had an impact on the generalisation of our findings. Furthermore, we did not classify LBW as per gestational age or classify individual cases in relation to the extent of prematurity and its related birth weight. An evaluation of the antenatal risk factors against this classification may be useful. The main strengths of this study were large sample size, the ability to identify modifiable risk factors for PB and LBW outcomes and use this information to promote health intervention programmes for pregnant women.

## Conclusion

This South African birth cohort study have identified maternal risk factors for PB, including cigarette smoking, middle age (> 30 years) pregnancies and low BMI. Maternal HIV infection and other sexually transmitted infections showed a trend towards being risk factors for PB and LBW outcomes. Our findings highlight the need for simple cost-effective intervention programs to educate women on the harmful effects of tobacco use, the optimal age for carrying pregnancies and the importance of proper weight gain during pregnancy. Routine testing women for sexually transmitted infections including tuberculosis during the antenatal period is necessary to minimise the risk of adverse birth outcomes.

## Supplementary information

**Additional file 1.**

## Data Availability

The datasets generated and analysed during the current study are not publicly available as our ethical approvals does not allow for this. However, should parties be interested in reviewing the data, the corresponding author will be able to approach the institutional ethical board to obtain necessary clearance, following reasonable request.

## Abbreviations

MACE: Mother and Child in the Environment Birth Cohort
LBW: Low birth weight
PB: Pre-term birth
AOR: Adjusted odds ratio
BMI: Body mass index
HIV: Human Immunodeficiency Virus
SGA: Small for gestational age
AIDS: Acquired Immunodeficiency Syndrome
KZN: KwaZulu-Natal province of South Africa
WHO: World Health Organisation
CI: Confidence Interval
UKZN: University of KwaZulu-Natal
BREC: Biomedical Research Ethics Committee (of UKZN)
ETS: Environmental tobacco smoke
ART: Anti-retroviral therapy
