# Supplementary material for: Maternal demographic and antenatal factors, low birth weight and preterm birth: findings from the mother and child in the environment (MACE) birth cohort, Durban, South Africa
Source: BMC Pregnancy Childbirth. 2020 Oct 16;20:628. doi: 10.1186/s12884-020-03328-6 (PMC7574237; doi:10.1186/s12884-020-03328-6)
Supplement: Supplementary file 1 — Additional file 1. [file 12884_2020_3328_MOESM1_ESM.pdf]

Mother and Child in the Environment (MACE)

1. Date:____/____/____ **2. Mother Identification No.** ☐☐☐ - ☐☐☐ - ☐☐☐
Day Month Year

Gestational Week ___________________

1. Name of respondent:	______________________<br>First<br>______________________<br>Middle<br>______________________<br>Surname
2. Phone numbers:	home: ________________<br>work: ________________<br>cell: __________________<br>other: ________________
3. What is your physical address?	_______________<br>House No.<br>_____________________________________<br>Road/Street<br>_____________________________________<br>City<br>______________________<br>Postal Code
4. How old are you?	_________years
5. What is your date of birth?	_____/_____/_____<br>day month year<br>☐9 Refused
6. What is your marital status?	☐1 Married<br>☐2 Living together<br>☐3 Single<br>☐4 Divorced<br>☐5 Separated<br>☐6 Widow<br>☐7 Other ______________

7. What is the highest grade or year of school you completed? **[READ CHOICES – select only one]**	☐1 Never attended school or only pre-school<br>☐2 Class 1 – Std 5 (Grades 1 through 7)<br>☐3 Std 6 – Std 9 (Grades 9 through 11- Some high school)<br>☐4 Std 10 / Matric (Grade 12 - High school graduate)<br>☐5 Non-degree training<br>☐6 College / technikon / university (1 year to 3 years)<br>☐7 Refused to answer
8. What is the highest grade or year of school your baby's father completed? **[READ CHOICES]**	☐1 Never attended school or only pre-school<br>☐2 Class 1 – Std 5 (Grades 1 through 7)<br>☐3 Std 6 – Std 9 (Grades 9 through 11- Some high school)<br>☐4 Std 10 / Matric (Grade 12 - High school graduate)<br>☐5 Non-degree training<br>☐6 College / technikon / university (1 year to 3 years)<br>☐7 Refused to answer

[INTRODUCTION: INTERVIEWER READS TO RESPONDENT]

The purpose of this questionnaire is to collect information about your pregnancy and reproductive health . If there is a question you do not want to answer, please let me know and we can skip it. All of your responses are confidential and will not shown to anyone outside the study team without your written consent. If you wish to stop the interview at anytime, please advise me. We can continue at a later time at your convenience

**A. HOUSEHOLD CONDITIONS**	
9. With whom do you live? *(Fill in one or several boxes.)*	☐1 Spouse<br>☐2 Partner<br>☐3 Parents<br>☐4 Parents-in-law<br>☐5 Children<br>☐6 No one<br>☐88 Others, describe _____________
10. How many people including you live in your home?	☐1 Number of people over 18 years<br>☐2 Number of people between 12 and 18 years<br>☐3 Number of people between 6 and 11 years<br>☐4 Number of people under 6 years
11. Usual language spoken at home:	☐1 English<br>☐2 Zulu<br>☐3 Xhosa<br>☐4 Afrikaans<br>☐88 Other<br>(Specify:_____________________)
12. How many of your children are at nursery school?	______<br>no. of children

13. What is your yearly gross income? (*Include child support, unemployment benefits and other allowances.)*	☐$_0$ No income<br>☐$_1$ Less than R2 000<br>☐$_2$ R10 001–30 000<br>☐$_3$ R30 001–75 000<br>☐$_4$ R75 001–150 000<br>☐$_5$ R150 001 and above<br>☐$_6$ Refused to answer
14. What is the baby's father's yearly gross income? (*Include child support, unemployment benefits and other allowances.)*	☐$_0$ No income<br>☐$_1$ Less than R2 000<br>☐$_2$ R10 001–30 000<br>☐$_3$ R30 001–75 000<br>☐$_4$ R75 001–150 000<br>☐$_5$ R150 001 and above<br>☐$_6$ Refused to answer
15. What type of housing do you live in?	☐$_1$ Detached house, Semidetached<br>☐$_2$ Farm<br>☐$_3$ Flat, Terraced flat, Apartment building<br>☐$_4$ Refused to answer<br>☐$_{88}$ Other ______________________
16. Has there been water damage, visible signs of fungus/mildew or a smell of mildew in your home in the past 3 months? *(Fill in one or several boxes.)*	☐$_1$ No<br>☐$_2$ Yes, water damage<br>☐$_3$ Yes, signs of fungus and mould<br>☐$_4$ Yes, a smell of mildew
17. What year was this house/structure originally built?	_____________<br>year<br>☐$_{99}$ don't know
**IF RESPONDENT IS UNSURE ASK**:<br>18. Would you say it was built:	☐$_1$ before 1970<br>☐$_2$ between 1970 and 1985<br>☐$_3$ after 1985
19. How many rooms are there in your home? (counting the kitchen, but not the bathroom or toilet)	_________________rooms
20 How long have you lived at this address?	____________ years<br>☐$_1$ less than 1 year
21. Is your home drinking water from the tap or from a river or dam?	☐$_1$ tap<br>☐$_2$ river or dam<br>☐$_3$ well<br>☐$_{88}$ other, please specify:<br>___________________________
22. Do any pets live in this home?	☐$_1$ Yes<br>☐$_2$ No **[If no, skip to Q24]**

23. What kind of pets live here?	
(a). a dog?	☐1 Yes<br>☐2 No
(b). a cat?	☐1 Yes<br>☐2 No
(c). a bird?	☐1 Yes<br>☐2 No
(d). any other pet(s)	☐1 Yes<br>☐2 No<br>☐88 please specify:<br>____________________
24. During the past 12 months was a room heater used to heat one or more rooms in this house?	☐1 Yes<br>☐2 No **[If no, skip to Q25]**<br>☐99 don't know **[If don't know, skip to Q25]**
(a). Was this heater fueled by	☐0 not applicable – no furnace<br>☐1 paraffin<br>☐2 gas<br>☐3 electricity<br>☐4 wood<br>☐5 coal<br>☐6 Gel<br>☐88 other, please specify:<br>___________________<br>☐88 don't know
25. During the past 12 months was one or more wood stoves used in this house?	☐1 Yes<br>☐2 No<br>☐99 Don't know
26. During the past 12 months was a fireplace used to heat the rooms in this house?	☐1 Yes<br>☐2 No<br>☐99 Don't know
27. During the past 12 months was the stove or oven ever used to heat this house?	☐1 Yes<br>☐2 No<br>☐99 Don't know
28. Is a stove or oven used for cooking in this house?	☐1 Yes<br>☐2 No<br>☐99 Don't know
(a). What is the primary source of heat for this stove or oven?	☐1 paraffin<br>☐2 gas<br>☐3 electricity<br>☐4 wood<br>☐5 coal<br>☐6 Gel<br>☐88 other, please specify:<br>___________________<br>☐99 don't know
**B. FAMILY SMOKING CHARACTERISTICS**	
29. Does anyone who lives here smoke cigarettes in the home?	☐1 Yes<br>☐2 No **[If no, skip to Q30]**
If yes, for each member of the household who smokes, please indicate	

Cigarettes smoked per day in the home			

**C. MENSTRUAL HISTORY**	
30. How old were you when you had your first menstrual period?	______________ years<br>age
31. How many days between each menstrual cycle?	☐$_{1}$ 28 days<br>☐$_{2}$ 35 days<br>☐$_{3}$ > 40 days<br>☐$_{99}$ don't know
32. Were your periods regular the year before you became pregnant?	☐$_{1}$ Yes<br>☐$_{2}$ No
33. During the last year before you became pregnant, did you miss your period for more than three months without being pregnant?	☐$_{1}$ Yes, due to another pregnancy<br>☐$_{2}$ Yes, due to other reasons<br>☐$_{3}$ No
34. Date of first day of last menstrual period	____/_____/20____<br>Day/month/year
35. Did your last menstrual period come at the expected time	☐$_{1}$ Yes<br>☐$_{2}$ No
36. Are you certain about the date of first day of last menstrual period?	☐$_{1}$ Certain<br>☐$_{2}$ Uncertain
37. Describe the duration, amount of bleeding and period pains of your last period	
(a). Was the duration	☐$_{1}$ Usual<br>☐$_{2}$ More than usual<br>☐$_{3}$ Less than usual
(b). Was the bleeding	☐$_{1}$ Usual<br>☐$_{2}$ More than usual<br>☐$_{3}$ Less than usual
(c). Was the pain	☐$_{1}$ Usual<br>☐$_{2}$ More than usual<br>☐$_{3}$ Less than usual
**D. CONTRACEPTION AND REPRODUCTIVE HISTORY**	
38. Have you/your partner at any time during the last year used the following methods to avoid becoming pregnant? *(Fill in all that apply.)*	☐$_{1}$ Condom<br>☐$_{2}$ Diaphragm<br>☐$_{3}$ IUD<br>☐$_{5}$ Hormone injection<br>☐$_{7}$ Pill<br>☐$_{11}$ No such methods<br>☐$_{88}$ Other
39. If you have used the pill, how long have you used them?	*Pill*<br>☐$_{0}$ never used<br>☐$_{1}$ Less than one year<br>☐$_{2}$ 1-3 years<br>☐$_{3}$ 4-6 years<br>☐$_{4}$ 7-9 years<br>☐$_{5}$ 10 years or more<br>**[If Pill never used, skip to Q43]**
40. If you have used the pill, how old were you when you first used it?	_______years old
41. Were you taking the pill during the last 4 months before this pregnancy?	☐$_{1}$ Yes<br>☐$_{2}$ No **[If no, skip to Q43]**
42. If yes, how long before your last menstrual period did you stop taking the pill	______ weeks
43. Was this pregnancy planned?	☐$_{1}$ Yes<br>☐$_{2}$ No**[If no, skip to Q44]**

(a). If yes, how many months did you have regular intercourse without contraception before you became pregnant?	☐1 Less than I month<br>☐2 1-2 months<br>☐3 3 months or more<br>☐4 Number of months if more than 3 ___________
44. Did you become pregnant even though you or your partner used contraceptives	☐1 Yes<br>☐2 No **[If no, skip to Q46]**
45. If yes, which type of contraceptives were used? *(Fill in all that apply.)*	☐1 Condom**[skip to Q47]**<br>☐2 Diaphragm**[skip to Q47]**<br>☐3 IUD<br>☐4 Hormone injection**[skip to Q47]**<br>☐5 Pill [**skip to Q47]**<br>☐88 Other ________________________
46. If you became pregnant while using an IUD, has it now been removed?	☐1 Yes<br>☐2 No
47. How long have you and the baby's father had a sexual relationship?	_________months or<br>___________years
48. How often did you have sexual intercourse during the four weeks before you became pregnant ?	☐1 Every day<br>☐2 5-6 times a week<br>☐3 3-4 times a week<br>☐4 1-2 times a week<br>☐5 1-2 times every two weeks<br>☐6 Less than 1-2 times every 2 weeks<br>☐7 Refused to answer
49. Have you ever been treated for infertility?	☐1 Yes<br>☐2 No **[If no, skip to Q52]**
50. If yes, was it in connection with this pregnancy or an earlier pregnancy?	☐1 This pregnancy<br>☐2 Previous pregnancy
51 (a) What type of treatment did you have?	☐1 Fallopian tube surgery<br>☐2 Other surgery<br>☐3 Medication for endometriosis<br>☐4 Hormone treatment<br>☐5 Insemination (injection of sperm)<br>☐6 Test-tube method<br>☐88 Other
52. Have you been given information about amniocentesis?	☐1 Yes<br>☐2 No
**E. PREVIOUS PREGNANCIES**	
53. Have you been pregnant before?<br>(Include all pregnancies that ended in abortion, miscarriage or stillbirth)	☐1 Yes<br>☐2 No **[If no, skip to Q62]**
**If yes, fill in for all earlier pregnancies.**<br>**[Include all pregnancies that ended in abortion, miscarriage or stillbirth as well as ectopic pregnancies].**	
54. Pregnancy No. 1	
(a). Year of pregnancy _______	__________<br>year
(b). Pregnancy outcome	☐1 Live infant born<br>☐2 Spontaneous abortion<br>☐3 Termination of pregnancy<br>☐4 Ectopic pregnancy
(c). Week of pregnancy for abortion/still birth	___________<br>weeks
(d) Did you breastfeed during this pregnancy?	☐1 Yes

	☐2 No [If No, skip to Q f)
(e). No. of months breast feeding	______________ months
(f). Weight gain during pregnancy	__________ kg
(g). Smoked during pregnancy	☐1 Yes<br>☐2 No
55. Pregnancy No. 2	
(a). Year of pregnancy _______	_________ year
(b). Pregnancy outcome	☐1 Live infant born<br>☐2 Spontaneous abortion<br>☐3 Termination of pregnancy<br>☐4 Ectopic pregnancy
(c). Week of pregnancy for abortion/still birth	__________ weeks
(d) Did you breastfeed during this pregnancy?	☐1 Yes<br>☐2 No [If No, skip to Q f)
(e). No. of months breast feeding	______________ months
(f). Weight gain during pregnancy	__________ kg
(g). Smoked during pregnancy	☐1 Yes<br>☐2 No
56. Pregnancy No. 3	
(a). Year of pregnancy _______	_________ year
(b). Pregnancy outcome	☐1 Live infant born<br>☐2 Spontaneous abortion<br>☐3 Termination of pregnancy<br>☐4 Ectopic pregnancy
(c). Week of pregnancy for abortion/still birth	__________ weeks
(d) Did you breastfeed during this pregnancy?	☐1 Yes<br>☐2 No [If No, skip to Q f)
(e). No. of months breast feeding	______________ months
(f). Weight gain during pregnancy	__________ kg
(g). Smoked during pregnancy	☐1 Yes<br>☐2 No
57. Pregnancy No. 4	
(a). Year of pregnancy _______	_________ year
(b). Pregnancy outcome	☐1 Live infant born<br>☐2 Spontaneous abortion<br>☐3 Termination of pregnancy<br>☐4 Ectopic pregnancy
(c). Week of pregnancy for abortion/still birth	__________ weeks
(d) Did you breastfeed during this pregnancy?	☐1 Yes<br>☐2 No [If No, skip to Q f)
(e). No. of months breast feeding	______________ months
(f). Weight gain during pregnancy	__________

	kg
(g). Smoked during pregnancy	☐1 Yes<br>☐2 No
58. Pregnancy No. 5	
(a). Year of pregnancy _______	__________<br>year
(b). Pregnancy outcome	☐1 Live infant born<br>☐2 Spontaneous abortion<br>☐3 Termination of pregnancy<br>☐4 Ectopic pregnancy
(c). Week of pregnancy for abortion/still birth	___________<br>weeks
(d) Did you breastfeed during this pregnancy?	☐1 Yes<br>☐2 No [If No, skip to Q f)
(e). No. of months breast feeding	__________________<br>months
(f). Weight gain during pregnancy	___________<br>kg
(g). Smoked during pregnancy	☐1 Yes<br>☐2 No
59. Pregnancy No. 6	
(a). Year of pregnancy _______	__________<br>year
(b). Pregnancy outcome	☐1 Live infant born<br>☐2 Spontaneous abortion<br>☐3 Termination of pregnancy<br>☐4 Ectopic pregnancy
(c). Week of pregnancy for abortion/still birth	___________<br>weeks
(d) Did you breastfeed during this pregnancy?	☐1 Yes<br>☐2 No [If No, skip to Q f)
(e). No. of months breast feeding	__________________<br>months
(f). Weight gain during pregnancy	___________<br>kg
(g). Smoked during pregnancy	☐1 Yes<br>☐2 No
60. Pregnancy No. 7	
(a). Year of pregnancy _______	__________<br>year
(b). Pregnancy outcome	☐1 Live infant born<br>☐2 Spontaneous abortion<br>☐3 Termination of pregnancy<br>☐4 Ectopic pregnancy
(c). Week of pregnancy for abortion/still birth	___________<br>weeks
(d) Did you breastfeed during this pregnancy?	☐1 Yes<br>☐2 No [If No, skip to Q f)
(e). No. of months breast feeding	__________________<br>months
(f). Weight gain during pregnancy	___________<br>kg
(g). Smoked during pregnancy	☐1 Yes<br>☐2 No
61. Pregnancy No. 8	
(a). Year of pregnancy _______	__________

	year
(b). Pregnancy outcome	☐1 Live infant born<br>☐2 Spontaneous abortion<br>☐3 Termination of pregnancy<br>☐4 Ectopic pregnancy
(c). Week of pregnancy for abortion/still birth	___________<br>weeks
(d) Did you breastfeed during this pregnancy?	☐1 Yes<br>☐2 No [If No, skip to Q f)
(e). No. of months breast feeding	_________________<br>months
(f). Weight gain during pregnancy	___________<br>kg
(g). Smoked during pregnancy	☐1 Yes<br>☐2 No
62. Have you had any of the following problems during previous pregnancies? *(Fill in all that apply.)*	
(c). Severe nausea and vomiting	☐1 Yes<br>☐2 No
(d). Eclampsia during pregnancy	☐1 Yes<br>☐2 No
(e). Diabetes during pregnancy	☐1 Yes<br>☐2 No
(f). Sugar in urine	☐1 Yes<br>☐2 No
(g). Problems with incontinence	☐1 Yes<br>☐2 No
	**NONE OF THE ABOVE**
**F. ILLNESSES AND HEALTH PROBLEMS DURING THIS PREGNANCY**	
63. Have you had bleeding from the vagina once or more during this pregnancy?	☐1 Yes<br>☐2 No **[If no, skip to Q69]**
64. Number of episodes:	____________
**If yes, describe from the first to the last bleeding episode. Give the date the bleeding started, how many days the bleeding lasted and how much you bled for each episode.**	
65. First Bleeding Episode:	
(a). Date when bleeding started	_______________
(b). No. of days bleeding	____________________
(c). Amount	☐1 Spotting<br>☐2 Light bleeding<br>☐3 Heavy bleeding
66. Second Bleeding Episode:	
(a). Date when bleeding started	_______________
(b). No. of days bleeding	____________________
(c). Amount	☐1 Spotting<br>☐2 Light bleeding<br>☐3 heavy bleeding
67. Third Bleeding Episode:	
(a). Date when bleeding started	_______________
(b). No. of days bleeding	____________________
(c). Amount	☐1 Spotting ☐2 Light bleeding<br>☐3 Heavy bleeding
68. Fourth Bleeding Episode:	
(a). Date when bleeding started	_______________
(b). No. of days bleeding	____________________

(c). Amount	☐1 Spotting ☐2 Light bleeding<br>☐3 Heavy bleeding
**Have you experienced any of the following illnesses or problems during this pregnancy? If you have used medication in connection with these problems give the name of the medicine, the weeks you took the medicines and how many days you took them.** ***(Include all types of medication, both prescription and over the counter medicines in addition to alternative and herbal remedies. Do not include vitamins and dietary supplements as these are discussed elsewhere.)***	
69. Abdominal Pain	☐1 Yes<br>☐2 No **[If no, skip to Q70]**
(a). Week of pregnancy	________ week of pregnancy
(b). Medication taken	☐1 Yes<br>☐2 No **[If no, skip to Q70]**
(i). Name of medication	__________
(ii). Do you have the bottle or package that you could show?<br>[Interview to indicate whether bottle or package seen:<br>Seen: Yes _____/No _____]	☐1 Yes<br>☐2 No
70. Back Pain	☐1 Yes<br>☐2 No **[If no, skip to Q71]**
(a). Week of pregnancy	________ week of pregnancy
(b). Medication taken	☐1 Yes<br>☐2 No **[If no, skip to Q71]**
(i). Name of medication	__________
(ii). Do you have the bottle or package that you could show?<br>[Interview to indicate whether bottle or package seen:<br>Seen: Yes _____/No _____]	☐1 Yes<br>☐2 No
(iii). Was this prescribed by:	☐1 Doctor<br>☐2 Clinic nurse<br>☐3 None of the above<br>☐4 Don't know
(iv). Was this over the counter medication	☐1 Yes<br>☐2 No
(v). No. of days taken	_________ days
71. Neck and Shoulder Pain	☐1 Yes<br>☐2 No **[If no, skip to Q72]**
(a). Week of pregnancy	________ week of pregnancy
(b). Medication taken	☐1 Yes<br>☐2 No **[If no, skip to Q72]**
(i). Name of medication	__________
(ii). Do you have the bottle or package that you could show?<br>[Interview to indicate whether bottle or package seen:<br>Seen: Yes _____/No _____]	☐1 Yes<br>☐2 No
(iii). Was this prescribed by:	☐1 Doctor<br>☐2 Clinic nurse<br>☐3 None of the above<br>☐4 Don't know
(iv). Was this over the counter medication	☐1 Yes

	☐$_2$ No
(v). No. of days taken	__________ days
72. Nausea	☐$_1$ Yes<br>☐$_2$ No **[If no, skip to Q73]**
(a). Week of pregnancy	_________ week of pregnancy
(b). Medication taken	☐$_1$ Yes<br>☐$_2$ No **[If no, skip to Q73]**
(i). Name of medication	__________
(ii). Do you have the bottle or package that you could show?<br>[Interview to indicate whether bottle or package seen: Seen: Yes _____/No _____]	☐$_1$ Yes<br>☐$_2$ No
(iii). Was this prescribed by:	☐$_1$ Doctor<br>☐$_2$ Clinic nurse<br>☐$_3$ None of the above<br>☐$_4$ Don't know
(iv). Was this over the counter medication	☐$_1$ Yes<br>☐$_2$ No
(v). No. of days taken	__________ days
73. Nausea with Vomiting	☐$_1$ Yes<br>☐$_2$ No **[If no, skip to Q74]**
(a). Week of pregnancy	_________ week of pregnancy
(b). Medication taken	☐$_1$ Yes<br>☐$_2$ No **[If no, skip to Q74]**
(i). Name of medication	__________
(ii). Do you have the bottle or package that you could show?<br>[Interview to indicate whether bottle or package seen: Seen: Yes _____/No _____]	☐$_1$ Yes<br>☐$_2$ No
(iii). Was this prescribed by:	☐$_1$ Doctor<br>☐$_2$ Clinic nurse<br>☐$_3$ None of the above<br>☐$_4$ Don't know
(iv). Was this over the counter medication	☐$_1$ Yes<br>☐$_2$ No
(v). No. of days taken	__________ days
74. Vaginal Thrush	☐$_1$ Yes<br>☐$_2$ No **[If no, skip to Q75]**
(a). Week of pregnancy	_________ week of pregnancy
(b). Medication taken	☐$_1$ Yes<br>☐$_2$ No **[If no, skip to Q75]**
(i). Name of medication	__________
(ii). Do you have the bottle or package that you could show?<br>[Interview to indicate whether bottle or package seen: Seen: Yes _____/No _____]	☐$_1$ Yes<br>☐$_2$ No
(iii). Was this prescribed by:	☐$_1$ Doctor<br>☐$_2$ Clinic nurse<br>☐$_3$ None of the above<br>☐$_4$ Don't know
(iv). Was this over the counter medication	☐$_1$ Yes<br>☐$_2$ No
(v). No. of days taken	__________ days

75. Vaginal Discharge	☐1 Yes<br>☐2 No **[If no, skip to Q76]**
(a). Week of pregnancy	________ week of pregnancy
(b). Medication taken	☐1 Yes<br>☐2 No **[If no, skip to Q76]**
(i). Name of medication	__________
(ii). Do you have the bottle or package that you could show?<br>[Interview to indicate whether bottle or package seen: Seen: Yes _____/No _____]	☐1 Yes<br>☐2 No
(iii). Was this prescribed by:	☐1 Doctor<br>☐2 Clinic nurse<br>☐3 None of the above<br>☐4 Don't know
(iv). Was this over the counter medication	☐1 Yes<br>☐2 No
(v). No. of days taken	_________ days
76. Itchy rash	☐1 Yes<br>☐2 No **[If no, skip to Q77]**
(a). Week of pregnancy	________ week of pregnancy
(b). Medication taken	☐1 Yes<br>☐2 No **[If no, skip to Q77]**
(i). Name of medication	__________
(ii). Do you have the bottle or package that you could show?<br>[Interview to indicate whether bottle or package seen: Seen: Yes _____/No _____]	☐1 Yes<br>☐2 No
(iii). Was this prescribed by:	☐1 Doctor<br>☐2 Clinic nurse<br>☐3 None of the above<br>☐4 Don't know
(iv). Was this over the counter medication	☐1 Yes<br>☐2 No
(v). No. of days taken	_________ days
77. Constipation	☐1 Yes<br>☐2 No **[If no, skip to Q78]**
(a). Week of pregnancy	________ week of pregnancy
(b). Medication taken	☐1 Yes<br>☐2 No **[If no, skip to Q78]**
(i). Name of medication	__________
(ii). Do you have the bottle or package that you could show?<br>[Interview to indicate whether bottle or package seen: Seen: Yes _____/No _____]	☐1 Yes<br>☐2 No
(iii). Was this prescribed by:	☐1 Doctor<br>☐2 Clinic nurse<br>☐3 None of the above<br>☐4 Don't know
(iv). Was this over the counter medication	☐1 Yes<br>☐2 No
(v). No. of days taken	_________ days
78. Diarrhoea	☐1 Yes

	☐2 No **[If no, skip to Q79]**
(a). Week of pregnancy	_________ week of pregnancy
(b). Medication taken	☐1 Yes<br>☐2 No **[If no, skip to Q79]**
(i). Name of medication	__________
(ii). Do you have the bottle or package that you could show?<br>[Interview to indicate whether bottle or package seen: Seen: Yes _____/No _____]	☐1 Yes<br>☐2 No
(iii). Was this prescribed by:	☐1 Doctor<br>☐2 Clinic nurse<br>☐3 None of the above<br>☐4 Don't know
(iv). Was this over the counter medication	☐1 Yes<br>☐2 No
(v). No. of days taken	__________ days
79. Unusual tiredness or sleepiness	☐1 Yes<br>☐2 No **[If no, skip to Q80]**
(a). Week of pregnancy	_________ week of pregnancy
(b). Medication taken	☐1 Yes<br>☐2 No **[If no, skip to Q80]**
(i). Name of medication	__________
(ii). Do you have the bottle or package that you could show?<br>[Interview to indicate whether bottle or package seen: Seen: Yes _____/No _____]	☐1 Yes<br>☐2 No
(iii). Was this prescribed by:	☐1 Doctor<br>☐2 Clinic nurse<br>☐3 None of the above<br>☐4 Don't know
(iv). Was this over the counter medication	☐1 Yes<br>☐2 No
(v). No. of days taken	__________ days
80. Sleeping Problems	☐1 Yes<br>☐2 No **[If no, skip to Q81]**
(a). Week of pregnancy	_________ week of pregnancy
(b). Medication taken	☐1 Yes<br>☐2 No **[If no, skip to Q81]**
(i). Name of medication	__________
(ii). Do you have the bottle or package that you could show?<br>[Interview to indicate whether bottle or package seen: Seen: Yes _____/No _____]	☐1 Yes<br>☐2 No
(iii). Was this prescribed by:	☐1 Doctor<br>☐2 Clinic nurse<br>☐3 None of the above<br>☐4 Don't know
(iv). Was this over the counter medication	☐1 Yes<br>☐2 No
(v). No. of days taken	__________ days
81. Heartburn	☐1 Yes<br>☐2 No **[If no, skip to Q82]**
(a). Week of pregnancy	_________ week of pregnancy

(b). Medication taken	☐1 Yes<br>☐2 No **[If no, skip to Q82]**
(i). Name of medication	__________
(ii). Do you have the bottle or package that you could show?<br>[Interview to indicate whether bottle or package seen: Seen: Yes _____/No _____]	☐1 Yes<br>☐2 No
(iii). Was this prescribed by:	☐1 Doctor<br>☐2 Clinic nurse<br>☐3 None of the above<br>☐4 Don't know
(iv). Was this over the counter medication	☐1 Yes<br>☐2 No
(v). No. of days taken	_________ days
82. Swelling of the legs and feet	☐1 Yes<br>☐2 No **[If no, skip to Q83]**
(a). Week of pregnancy	________ week of pregnancy
(b). Medication taken	☐1 Yes<br>☐2 No **[If no, skip to Q83]**
(i). Name of medication	__________
(ii). Do you have the bottle or package that you could show?<br>[Interview to indicate whether bottle or package seen: Seen: Yes _____/No _____]	☐1 Yes<br>☐2 No
(iii). Was this prescribed by:	☐1 Doctor<br>☐2 Clinic nurse<br>☐3 None of the above<br>☐4 Don't know
(iv). Was this over the counter medication	☐1 Yes<br>☐2 No
(v). No. of days taken	_________ days
83. Fever with rash	☐1 Yes<br>☐2 No **[If no, skip to Q84]**
(a). Week of pregnancy	________ week of pregnancy
(b). Medication taken	☐1 Yes<br>☐2 No **[If no, skip to Q84]**
(i). Name of medication	__________
(ii). Do you have the bottle or package that you could show?<br>[Interview to indicate whether bottle or package seen: Seen: Yes _____/No _____]	☐1 Yes<br>☐2 No
(iii). Was this prescribed by:	☐1 Doctor<br>☐2 Clinic nurse<br>☐3 None of the above<br>☐4 Don't know
(iv). Was this over the counter medication	☐1 Yes<br>☐2 No
(v). No. of days taken	_________ days
84. High fever (over 38.5 degrees C)	☐1 Yes<br>☐2 No **[If no, skip to Q85]**
(a). Week of pregnancy	________ week of pregnancy
(b). Medication taken	☐1 Yes<br>☐2 No **[If no, skip to Q85]**

(i). Name of medication	__________
(ii). Do you have the bottle or package that you could show?<br>[Interview to indicate whether bottle or package seen: Seen: Yes _____/No _____]	☐1 Yes<br>☐2 No
(iii). Was this prescribed by:	☐1 Doctor<br>☐2 Clinic nurse<br>☐3 None of the above<br>☐4 Don't know
(iv). Was this over the counter medication	☐1 Yes<br>☐2 No
(v). No. of days taken	__________ days
85. Common cold	☐1 Yes<br>☐2 No **[If no, skip to Q 86]**
(a). Week of pregnancy	_________ week of pregnancy
(b). Medication taken	☐1 Yes<br>☐2 No **[If no, skip to Q86]**
(i). Name of medication	__________
(ii). Do you have the bottle or package that you could show?<br>[Interview to indicate whether bottle or package seen: Seen: Yes _____/No _____]	☐1 Yes<br>☐2 No
(iii). Was this prescribed by:	☐1 Doctor<br>☐2 Clinic nurse<br>☐3 None of the above<br>☐4 Don't know
(iv). Was this over the counter medication	☐1 Yes<br>☐2 No
(v). No. of days taken	__________ days
86. Throat infection	☐1 Yes<br>☐2 No **[If no, skip to Q87]**
(a). Week of pregnancy	_________ week of pregnancy
(b). Medication taken	☐1 Yes<br>☐2 No **[If no, skip to Q87]**
(i). Name of medication	__________
(ii). Do you have the bottle or package that you could show?<br>[Interview to indicate whether bottle or package seen: Seen: Yes _____/No _____]	☐1 Yes<br>☐2 No
(iii). Was this prescribed by:	☐1 Doctor<br>☐2 Clinic nurse<br>☐3 None of the above<br>☐4 Don't know
(iv). Was this over the counter medication	☐1 Yes<br>☐2 No
(v). No. of days taken	__________ days
87. Sinusitus with ear infection	☐1 Yes<br>☐2 No **[If no, skip to Q88]**
(a). Week of pregnancy	_________ week of pregnancy
(b). Medication taken	☐1 Yes<br>☐2 No **[If no, skip to Q88**
(i). Name of medication	__________

(ii). Do you have the bottle or package that you could show?<br>[Interview to indicate whether bottle or package seen: Seen: Yes _____/No _____]	☐1 Yes<br>☐2 No
(iii). Was this prescribed by:	☐1 Doctor<br>☐2 Clinic nurse<br>☐3 None of the above<br>☐4 Don't know
(iv). Was this over the counter medication	☐1 Yes<br>☐2 No
(v). No. of days taken	__________ days
88. The 'Flu	☐1 Yes<br>☐2 No **[If no, skip to Q89]**
(a). Week of pregnancy	_________ week of pregnancy
(b). Medication taken	☐1 Yes<br>☐2 No **[If no, skip to Q89]**
(i). Name of medication	__________
(ii). Do you have the bottle or package that you could show?<br>[Interview to indicate whether bottle or package seen: Seen: Yes _____/No _____]	☐1 Yes<br>☐2 No
(iii). Was this prescribed by:	☐1 Doctor<br>☐2 Clinic nurse<br>☐3 None of the above<br>☐4 Don't know
(iv). Was this over the counter medication	☐1 Yes<br>☐2 No
(v). No. of days taken	__________ days
89. Pneumonia or bronchitis	☐1 Yes<br>☐2 No **[If no, skip to Q90]**
(a). Week of pregnancy	_________ week of pregnancy
(b). Medication taken	☐1 Yes<br>☐2 No **[If no, skip to Q90]**
(i). Name of medication	__________
(ii). Do you have the bottle or package that you could show?<br>[Interview to indicate whether bottle or package seen: Seen: Yes _____/No _____]	☐1 Yes<br>☐2 No
(iii). Was this prescribed by:	☐1 Doctor<br>☐2 Clinic nurse<br>☐3 None of the above<br>☐4 Don't know
(iv). Was this over the counter medication	☐1 Yes<br>☐2 No
(v). No. of days taken	__________ days
90. Sugar in the urine	☐1 Yes<br>☐2 No **[If no, skip to Q91]**
(a). Week of pregnancy	_________ week of pregnancy
(b). Medication taken	☐1 Yes<br>☐2 No **[If no, skip to Q91]**
(i). Name of medication	__________
(ii). Do you have the bottle or package that you could show?	☐1 Yes<br>☐2 No

[Interview to indicate whether bottle or package seen:<br>Seen: Yes _____/No _____]	
(iii). Was this prescribed by:	☐1 Doctor<br>☐2 Clinic nurse<br>☐3 None of the above<br>☐4 Don't know
(iv). Was this over the counter medication	☐1 Yes<br>☐2 No
(v). No. of days taken	_________ days
91. Protein in the urine	☐1 Yes<br>☐2 No **[If no, skip to Q92]**
(a). Week of pregnancy	________ week of pregnancy
(b). Medication taken	☐1 Yes<br>☐2 No **[If no, skip to Q92]**
(i). Name of medication	_________
(ii). Do you have the bottle or package that you could show?<br>[Interview to indicate whether bottle or package seen:<br>Seen: Yes _____/No _____]	☐1 Yes<br>☐2 No
(iii). Was this prescribed by:	☐1 Doctor<br>☐2 Clinic nurse<br>☐3 None of the above<br>☐4 Don't know
(iv). Was this over the counter medication	☐1 Yes<br>☐2 No
(v). No. of days taken	_________ days
92. In the last three months, have you had diarrhea that lasted for more than three days?	☐1 Yes<br>☐2 No
93. In the last three months did you have fever for more than one month on end?	☐1 Yes<br>☐2 No
94. Have you had white sores in your mouth over the last three months?	☐1 Yes<br>☐2 No
95. Do you have swollen lymph nodes in your neck, under your arms or in your groin?	☐1 Yes<br>☐2 No
96. Have you had shingles (painful blisters or sores usually in a narrow band on one side of the head or body) over the last 12 months?	☐1 Yes<br>☐2 No

G. PAST AND PRESENT ILLNESSES AND HEALTH PROBLEMS	
**Do you have or have you had any of the following illnesses or health problems? If you have taken medication (tablets, mixtures, suppositories, inhalers, creams, etc.) in conjunction with the illness or health problem give the name(s) of the medication(s) and when you took them.**	
**Respiratory, Allergy and Skin Disorders**	
97. Have you ever been told by a doctor that you have asthma	☐1 Yes<br>☐2 No [If no, skip to Q98]
(a). Were you told this more than six months before this pregnancy?	☐1 Yes [If yes, skip to d]<br>☐2 No
(b). Were you told this in the last six months before this pregnancy?	☐1 Yes [If yes, skip to d]<br>☐2 No
(c). Were you told this during this pregnancy?	☐1 Yes<br>☐2 No
(i). If yes, in what week of the pregnancy?	☐1 0-4 weeks<br>☐2 5-8 weeks<br>☐3 9-12 weeks<br>☐4 13+ weeks
(d). Have you taken any medication for this problem	☐1 Yes<br>☐2 No [If no, skip to Q98]
(i). Name of medication	__________
(ii). Do you have the bottle or package that you could show?<br>[Interview to indicate whether bottle or package seen:<br>Seen: Yes _____/No _____]	☐1 Yes<br>☐2 No
(iii). Was this prescribed by:	☐1 Doctor<br>☐2 Clinic nurse<br>☐3 None of the above<br>☐4 Don't know
(iv). Was this over the counter medication	☐1 Yes<br>☐2 No
(v). No. of days taken	__________ days
98. Have you ever been told by a doctor that you have chronic bronchitis	☐1 Yes<br>☐2 No [If no, skip to Q99]
(a). Were you told this more than six months before this pregnancy?	☐1 Yes<br>☐2 No
(b). Were you told this in the last six months before this pregnancy?	☐1 Yes<br>☐2 No
(c). Were you told this during this pregnancy?	☐1 Yes<br>☐2 No
(i). If yes, in what week of the pregnancy?	☐1 0-4 weeks<br>☐2 5-8 weeks<br>☐3 9-12 weeks<br>☐4 13+ weeks
(d). Have you taken any medication for this problem	☐1 Yes<br>☐2 No [If no, skip to Q99]
(i). Name of medication	__________
(ii). Do you have the bottle or package that you could show?<br>[Interview to indicate whether bottle or package seen:<br>Seen: Yes _____/No _____]	☐1 Yes<br>☐2 No
(iii). Was this prescribed by:	☐1 Doctor<br>☐2 Clinic nurse<br>☐3 None of the above

	☐4 Don't know
(iv). Was this over the counter medication	☐1 Yes<br>☐2 No
(v). No. of days taken	_________ days
99. Have you ever been told by a doctor that you have emphysema	☐1 Yes<br>☐2 No [If no, skip to Q100]
(a). Were you told this more than six months before this pregnancy?	☐1 Yes<br>☐2 No
(b). Were you told this in the last six months before this pregnancy?	☐1 Yes<br>☐2 No
(c). Were you told this during this pregnancy?	☐1 Yes<br>☐2 No
(i). If yes, in what week of the pregnancy?	☐1 0-4 weeks<br>☐2 5-8 weeks<br>☐3 9-12 weeks<br>☐4 13+ weeks
(d). Have you taken any medication for this problem	☐1 Yes<br>☐2 No [If no, skip to Q100]
(i). Name of medication	_________
(ii). Do you have the bottle or package that you could show?<br>[Interview to indicate whether bottle or package seen:<br>Seen: Yes _____/No _____]	☐1 Yes<br>☐2 No
(iii). Was this prescribed by:	☐1 Doctor<br>☐2 Clinic nurse<br>☐3 None of the above<br>☐4 Don't know
(iv). Was this over the counter medication	☐1 Yes<br>☐2 No
(v). No. of days taken	_________ days
100. Have you ever been told by a doctor that you have TB (or tuberculosis)	☐1 Yes<br>☐2 No [If no, skip to Q101]
(a). Were you told this more than six months before this pregnancy?	☐1 Yes<br>☐2 No
(b). Were you told this in the last six months before this pregnancy?	☐1 Yes<br>☐2 No
(c). Were you told this during this pregnancy?	☐1 Yes<br>☐2 No
(i). If yes, in what week of the pregnancy?	☐1 0-4 weeks<br>☐2 5-8 weeks<br>☐3 9-12 weeks<br>☐4 13+ weeks
(d). Have you taken any medication for this problem	☐1 Yes<br>☐2 No [If no, skip to Q101]
(i). Name of medication	_________
(ii). Do you have the bottle or package that you could show?<br>[Interview to indicate whether bottle or package seen:<br>Seen: Yes _____/No _____]	☐1 Yes<br>☐2 No
(iii). Was this prescribed by:	☐1 Doctor<br>☐2 Clinic nurse<br>☐3 None of the above

	☐4 Don't know
(iv). Was this over the counter medication	☐1 Yes<br>☐2 No
(v). No. of days taken	__________ days
101. Have you ever been told by a doctor that you have hayfever?	☐1 Yes<br>☐2 No **[If no, skip to Q102]**
(a). Were you told this more than six months before this pregnancy?	☐1 Yes<br>☐2 No
(b). Were you told this in the last six months before this pregnancy?	☐1 Yes<br>☐2 No
(c). Were you told this during this pregnancy?	☐1 Yes<br>☐2 No
(i). If yes, in what week of the pregnancy?	☐1 0-4 weeks<br>☐2 5-8 weeks<br>☐3 9-12 weeks<br>☐4 13+ weeks
(d). Have you taken any medication for this problem	☐1 Yes<br>☐2 No **[If no, skip to Q102]**
(i). Name of medication	__________
(ii). Do you have the bottle or package that you could show?<br>[Interview to indicate whether bottle or package seen:<br>Seen: Yes _____/No _____]	☐1 Yes<br>☐2 No
(iii). Was this prescribed by:	☐1 Doctor<br>☐2 Clinic nurse<br>☐3 None of the above<br>☐4 Don't know
(iv). Was this over the counter medication	☐1 Yes<br>☐2 No
(v). No. of days taken	__________ days
102. Have you ever been told by a doctor that you have an allergy?	☐1 Yes<br>☐2 No **[If no, skip to Q103]**
(a). Were you told this more than six months before this pregnancy?	☐1 Yes<br>☐2 No
(b). Were you told this in the last six months before this pregnancy?	☐1 Yes<br>☐2 No
(c). Were you told this during this pregnancy?	☐1 Yes<br>☐2 No
(i). If yes, in what week of the pregnancy?	☐1 0-4 weeks<br>☐2 5-8 weeks<br>☐3 9-12 weeks<br>☐4 13+ weeks
(d). Have you taken any medication for this problem	☐1 Yes<br>☐2 No **[If no, skip to Q103]**
(i). Name of medication	__________
(ii). Do you have the bottle or package that you could show?<br>[Interview to indicate whether bottle or package seen:<br>Seen: Yes _____/No _____]	☐1 Yes<br>☐2 No
(iii). Was this prescribed by:	☐1 Doctor<br>☐2 Clinic nurse<br>☐3 None of the above

	☐4 Don't know
(iv). Was this over the counter medication	☐1 Yes<br>☐2 No
(v). No. of days taken	__________ days
(e). What were you told you were allergic too?	☐1 food<br>☐2 pollen<br>☐3 animal hair<br>☐4 medication<br>☐88 other: ________________
103. Have you ever been told by a doctor that you have one or more of the following skin problems	☐1 Eczema<br>☐2 Urticaria (hives)<br>☐3 Psoriasis<br>☐4 Cold sores (herpes)<br>☐5 Acne<br>☐6 Other: ____________________<br>☐7 No **[If no, skip to Q104]**
(a). Were you told this more than six months before this pregnancy?	☐1 Yes<br>☐2 No
(b). Were you told this in the last six months before this pregnancy?	☐1 Yes<br>☐2 No
(c). Were you told this during this pregnancy?	☐1 Yes<br>☐2 No
(i). If yes, in what week of the pregnancy?	☐1 0-4 weeks<br>☐2 5-8 weeks<br>☐3 9-12 weeks<br>☐4 13+ weeks
(d). Have you taken any medication for this problem	☐1 Yes<br>☐2 No **[If no, skip to Q104]**
(i). Name of medication	__________ (skin disease: _________)<br>__________ (skin disease: _________)<br>__________ (skin disease: _________)<br>__________ (skin disease: _________)<br>__________ (skin disease: _________)<br>__________ (skin disease: _________)<br>__________ (skin disease: _________)
(ii). Do you have the bottle or package that you could show?<br>[Interview to indicate whether bottle or package seen:<br>Seen: Yes _____/No _____]	☐1 Yes<br>☐2 No
(iii). Was this prescribed by:	☐1 Doctor<br>☐2 Clinic nurse<br>☐3 None of the above<br>☐4 Don't know
(iv). Was this over the counter medication	☐1 Yes<br>☐2 No
(v). No. of days taken	__________ days
104. Have you ever been told by a doctor that you have diabetes	☐1 Yes<br>☐2 No **[If no, skip to Q105]**
(a). Were you told this more than six months before this pregnancy?	☐1 Yes<br>☐2 No
(i). If yes, was this in a previous pregnancy?	☐1 Yes<br>☐2 No

(ii) If yes, was your blood sugar normal after the pregnancy?	☐1 Yes<br>☐2 No
(b). Were you told this in the last six months before this pregnancy?	☐1 Yes<br>☐2 No
(c). Were you told this during this pregnancy?	☐1 Yes<br>☐2 No
(i). If yes, in what week of the pregnancy?	☐1 0-4 weeks<br>☐2 5-8 weeks<br>☐3 9-12 weeks<br>☐4 13+ weeks
(ii). Are you <u>now</u> taking insulin?	☐1 Yes<br>☐2 No
(iii). Was this prescribed by:	☐1 Doctor<br>☐2 Clinic nurse<br>☐3 None of the above<br>☐4 Don't know
(iv). Name of medication	__________
(v). Do you have the bottle or package that you could show?<br>[Interview to indicate whether bottle or package seen:<br>Seen: Yes _____/No _____]	☐1 Yes<br>☐2 No
(vi). Was this prescribed by a doctor	☐1 Yes<br>☐2 No
(vii). Was this over the counter medication	☐1 Yes<br>☐2 No
(viii). No. of days taken	_________ days
**Heart / Blood / Metabolism / Blood vessels**	
105. Have you ever been told by a doctor that you have heart defect since birth (a congenital heart defect)?	☐1 Yes<br>☐2 No **[If no, skip to Q106]**
(a). Were you told this more than six months before this pregnancy?	☐1 Yes<br>☐2 No
(b). Were you told this in the last six months before this pregnancy?	☐1 Yes<br>☐2 No
(c). Were you told this during this pregnancy?	☐1 Yes<br>☐2 No
(i). If yes, in what week of the pregnancy?	☐1 0-4 weeks<br>☐2 5-8 weeks<br>☐3 9-12 weeks<br>☐4 13+ weeks
(d). Have you taken any medication for this problem	☐1 Yes<br>☐2 No **[If no, skip to Q106]**
(i). Name of medication	__________
(ii). Do you have the bottle or package that you could show?<br>[Interview to indicate whether bottle or package seen:<br>Seen: Yes _____/No _____]	☐1 Yes<br>☐2 No
(iii). Was this prescribed by:	☐1 Doctor<br>☐2 Clinic nurse<br>☐3 None of the above<br>☐4 Don't know
(iv). Was this over the counter medication	☐1 Yes<br>☐2 No
(v). No. of days taken	_________ days

106. Have you ever been told by a doctor that you have any other heart disease	☐1 Yes<br>☐2 No **[If no, skip to Q107]**
(a). If yes, please describe	___________________
(b). Were you told this more than six months before this pregnancy?	☐1 Yes<br>☐2 No
(c). Were you told this in the last six months before this pregnancy?	☐1 Yes<br>☐2 No
(d). Were you told this during this pregnancy?	☐1 Yes<br>☐2 No
(i). If yes, in what week of the pregnancy?	☐1 0-4 weeks<br>☐2 5-8 weeks<br>☐3 9-12 weeks<br>☐4 13+ weeks
(e). Have you taken any medication for this problem	☐1 Yes<br>☐2 No **[If no, skip to Q107]**
(i). Name of medication	__________
(ii). Do you have the bottle or package that you could show?<br>[Interview to indicate whether bottle or package seen:<br>Seen: Yes _____/No _____]	☐1 Yes<br>☐2 No
(iii). Was this prescribed by:	☐1 Doctor<br>☐2 Clinic nurse<br>☐3 None of the above<br>☐4 Don't know
(iv). Was this over the counter medication	☐1 Yes<br>☐2 No
(v). No. of days taken	__________ days
107. Have you ever been told by a doctor that you have high cholesterol	☐1 Yes<br>☐2 No **[If no, skip to Q108]**
(a). Were you told this more than six months before this pregnancy?	☐1 Yes<br>☐2 No
(b). Were you told this in the last six months before this pregnancy?	☐1 Yes<br>☐2 No
(c). Were you told this during this pregnancy?	☐1 Yes<br>☐2 No
(i). If yes, in what week of the pregnancy?	☐1 0-4 weeks<br>☐2 5-8 weeks<br>☐3 9-12 weeks<br>☐4 13+ weeks
(d). Have you taken any medication for this problem	☐1 Yes<br>☐2 No **[If no, skip to Q108]**
(i). Name of medication	__________
(ii). Do you have the bottle or package that you could show?<br>[Interview to indicate whether bottle or package seen:<br>Seen: Yes _____/No _____]	☐1 Yes<br>☐2 No
(iii). Was this prescribed by:	☐1 Doctor<br>☐2 Clinic nurse<br>☐3 None of the above<br>☐4 Don't know
(iv). Was this over the counter medication	☐1 Yes<br>☐2 No
(v). No. of days taken	__________ days

108. Have you ever been told by a doctor that you have high blood pressure?	☐1 Yes<br>☐2 No **[If no, skip to Q109]**
(a). Were you told this more than six months before this pregnancy?	☐1 Yes<br>☐2 No
(i). If yes, was this in a previous pregnancy?	☐1 Yes<br>☐2 No
(ii). If yes, was your blood pressure normal after the pregnancy?	☐1 Yes<br>☐2 No
(b). Were you told this in the last six months before this pregnancy?	☐1 Yes<br>☐2 No
(c). Were you told this during this pregnancy?	☐1 Yes<br>☐2 No
(i). If yes, in what week of the pregnancy?	☐1 0-4 weeks<br>☐2 5-8 weeks<br>☐3 9-12 weeks<br>☐4 13+ weeks
(d). Have you taken any medication for this problem	☐1 Yes<br>☐2 No **[If no, skip to Q109]**
(i). Name of medication	__________
(ii). Do you have the bottle or package that you could show?<br>[Interview to indicate whether bottle or package seen:<br>Seen: Yes _____/No _____]	☐1 Yes<br>☐2 No
(iii). Was this prescribed by:	☐1 Doctor<br>☐2 Clinic nurse<br>☐3 None of the above<br>☐4 Don't know
(iv). Was this over the counter medication	☐1 Yes<br>☐2 No
(v). No. of days taken	__________ days
109. Have you ever been told by a doctor that you have hypothyroidism or hyperthyroidism	☐1 Yes<br>☐2 No **[If no, skip to Q110]**
(a). Were you told this more than six months before this pregnancy?	☐1 Yes<br>☐2 No
(b). Were you told this in the last six months before this pregnancy?	☐1 Yes<br>☐2 No
(c). Were you told this during this pregnancy?	☐1 Yes<br>☐2 No
(i). If yes, in what week of the pregnancy?	☐1 0-4 weeks<br>☐2 5-8 weeks<br>☐3 9-12 weeks<br>☐4 13+ weeks
(d). Have you taken any medication for this problem	☐1 Yes<br>☐2 No **[If no, skip to Q110]**
(i). Name of medication	__________
(ii). Do you have the bottle or package that you could show?<br>[Interview to indicate whether bottle or package seen:<br>Seen: Yes _____/No _____]	☐1 Yes<br>☐2 No
(iii). Was this prescribed by:	☐1 Doctor<br>☐2 Clinic nurse<br>☐3 None of the above

	☐4 Don't know
(iv). Was this over the counter medication	☐1 Yes<br>☐2 No
(v). No. of days taken	__________ days
110. Have you ever been told by a doctor that you have anaemia	☐1 Yes<br>☐2 No **[If no, skip to Q111]**
(a). Were you told this more than six months before this pregnancy?	☐1 Yes<br>☐2 No
(b). Were you told this in the last six months before this pregnancy?	☐1 Yes<br>☐2 No
(c). Were you told this during this pregnancy?	☐1 Yes<br>☐2 No
(i). If yes, in what week of the pregnancy?	☐1 0-4 weeks<br>☐2 5-8 weeks<br>☐3 9-12 weeks<br>☐4 13+ weeks
(d). Have you taken any medication for this problem	☐1 Yes<br>☐2 No **[If no, skip to Q111]**
(i). Name of medication	__________
(ii). Do you have the bottle or package that you could show?<br>[Interview to indicate whether bottle or package seen:<br>Seen: Yes _____/No _____]	☐1 Yes<br>☐2 No
(iii). Was this prescribed by:	☐1 Doctor<br>☐2 Clinic nurse<br>☐3 None of the above<br>☐4 Don't know
(iv). Was this over the counter medication	☐1 Yes<br>☐2 No
(v). No. of days taken	__________ days
111. Have you ever been told by a doctor that you have B-12/folic acid insufficiency	☐1 Yes<br>☐2 No **[If no, skip to Q112]**
(a). Were you told this more than six months before this pregnancy?	☐1 Yes<br>☐2 No
(b). Were you told this in the last six months before this pregnancy?	☐1 Yes<br>☐2 No
(c). Were you told this during this pregnancy?	☐1 Yes<br>☐2 No
(i). If yes, in what week of the pregnancy?	☐1 0-4 weeks<br>☐2 5-8 weeks<br>☐3 9-12 weeks<br>☐4 13+ weeks
(d). Have you taken any medication for this problem	☐1 Yes<br>☐2 No **[If no, skip to Q112]**
(i). Name of medication	__________
(ii). Do you have the bottle or package that you could show?<br>[Interview to indicate whether bottle or package seen:<br>Seen: Yes _____/No _____]	☐1 Yes<br>☐2 No
(iii). Was this prescribed by:	☐1 Doctor<br>☐2 Clinic nurse<br>☐3 None of the above

	☐4 Don't know
(iv). Was this over the counter medication	☐1 Yes<br>☐2 No
(v). No. of days taken	__________ days
**Gastrointestinal**	
112. Have you ever been told by a doctor that you have Hepatitis/jaundice/	☐1 Yes<br>☐2 No **[If no, skip to Q113]**
(a). Were you told this more than six months before this pregnancy?	☐1 Yes<br>☐2 No
(b). Were you told this in the last six months before this pregnancy?	☐1 Yes<br>☐2 No
(c). Were you told this during this pregnancy?	☐1 Yes<br>☐2 No
(i). If yes, in what week of the pregnancy?	☐1 0-4 weeks<br>☐2 5-8 weeks<br>☐3 9-12 weeks<br>☐4 13+ weeks
(d). Have you taken any medication for this problem	☐1 Yes<br>☐2 No **[If no, skip to Q113]**
(i). Name of medication	__________
(ii). Do you have the bottle or package that you could show?<br>[Interview to indicate whether bottle or package seen:<br>Seen: Yes _____/No _____]	☐1 Yes<br>☐2 No
(iii). Was this prescribed by:	☐1 Doctor<br>☐2 Clinic nurse<br>☐3 None of the above<br>☐4 Don't know
(iv). Was this over the counter medication	☐1 Yes<br>☐2 No
(v). No. of days taken	__________ days
113. Have you ever been told by a doctor that you have Gall stones	☐1 Yes<br>☐2 No **[If no, skip to Q114]**
(a). Were you told this more than six months before this pregnancy?	☐1 Yes<br>☐2 No
(b). Were you told this in the last six months before this pregnancy?	☐1 Yes<br>☐2 No
(c). Were you told this during this pregnancy?	☐1 Yes<br>☐2 No
(i). If yes, in what week of the pregnancy?	☐1 0-4 weeks<br>☐2 5-8 weeks<br>☐3 9-12 weeks<br>☐4 13+ weeks
(d). Have you taken any medication for this problem	☐1 Yes<br>☐2 No **[If no, skip to Q114]**
(i). Name of medication	__________
(ii). Do you have the bottle or package that you could show?<br>[Interview to indicate whether bottle or package seen:<br>Seen: Yes _____/No _____]	☐1 Yes<br>☐2 No
(iii). Was this prescribed by:	☐1 Doctor<br>☐2 Clinic nurse<br>☐3 None of the above

	☐4 Don't know
(iv). Was this over the counter medication	☐1 Yes<br>☐2 No
(v). No. of days taken	_________ days
114. Have you ever been told by a doctor that you have stomach ulcer	☐1 Yes<br>☐2 No **[If no, skip to Q115]**
(a). Were you told this more than six months before this pregnancy?	☐1 Yes<br>☐2 No
(b). Were you told this in the last six months before this pregnancy?	☐1 Yes<br>☐2 No
(c). Were you told this during this pregnancy?	☐1 Yes<br>☐2 No
(i). If yes, in what week of the pregnancy?	☐1 0-4 weeks<br>☐2 5-8 weeks<br>☐3 9-12 weeks<br>☐4 13+ weeks
(d). Have you taken any medication for this problem	☐1 Yes<br>☐2 No **[If no, skip to Q115]**
(i). Name of medication	__________
(ii). Do you have the bottle or package that you could show?<br>[Interview to indicate whether bottle or package seen:<br>Seen: Yes _____/No _____]	☐1 Yes<br>☐2 No
(iii). Was this prescribed by:	☐1 Doctor<br>☐2 Clinic nurse<br>☐3 None of the above<br>☐4 Don't know
(iv). Was this over the counter medication	☐1 Yes<br>☐2 No
(v). No. of days taken	_________ days
115. Have you ever been told by a doctor that you have other gastro-intestinal problems	☐1 Yes<br>☐2 No **[If no, skip to Q116]**
(a). If yes, please describe	____________________
(b). Were you told this more than six months before this pregnancy?	☐1 Yes<br>☐2 No
(c). Were you told this in the last six months before this pregnancy?	☐1 Yes<br>☐2 No
(d). Were you told this during this pregnancy?	☐1 Yes<br>☐2 No
(i). If yes, in what week of the pregnancy?	☐1 0-4 weeks<br>☐2 5-8 weeks<br>☐3 9-12 weeks<br>☐4 13+ weeks
(e). Have you taken any medication for this problem	☐1 Yes<br>☐2 No **[If no, skip to Q116]**
(i). Name of medication	__________
(ii). Do you have the bottle or package that you could show?<br>[Interview to indicate whether bottle or package seen:<br>Seen: Yes _____/No _____]	☐1 Yes<br>☐2 No
(iii). Was this prescribed by:	☐1 Doctor<br>☐2 Clinic nurse

	☐3 None of the above<br>☐4 Don't know
(iv). Was this over the counter medication	☐1 Yes<br>☐2 No
(v). No. of days taken	__________ days
**Musculoskeletal or Connective tissue**	
116. Have you ever been told by a doctor that you have Rheumatoid arthritis/	☐1 Yes<br>☐2 No **[If no, skip to Q117]**
(a). Were you told this more than six months before this pregnancy?	☐1 Yes<br>☐2 No
(b). Were you told this in the last six months before this pregnancy?	☐1 Yes<br>☐2 No
(c). Were you told this during this pregnancy?	☐1 Yes<br>☐2 No
(i). If yes, in what week of the pregnancy?	☐1 0-4 weeks<br>☐2 5-8 weeks<br>☐3 9-12 weeks<br>☐4 13+ weeks
(d). Have you taken any medication for this problem	☐1 Yes<br>☐2 No **[If no, skip to Q117]**
(i). Name of medication	__________
(ii). Do you have the bottle or package that you could show?<br>[Interview to indicate whether bottle or package seen:<br>Seen: Yes _____/No _____]	☐1 Yes<br>☐2 No
(iii). Was this prescribed by:	☐1 Doctor<br>☐2 Clinic nurse<br>☐3 None of the above<br>☐4 Don't know
(iv). Was this over the counter medication	☐1 Yes<br>☐2 No
(v). No. of days taken	__________ days
117. Have you ever been told by a doctor that you have Lupus (SLE)/	☐1 Yes<br>☐2 No **[If no, skip to Q118]**
(a). Were you told this more than six months before this pregnancy?	☐1 Yes<br>☐2 No
(b). Were you told this in the last six months before this pregnancy?	☐1 Yes<br>☐2 No
(c). Were you told this during this pregnancy?	☐1 Yes<br>☐2 No
(i). If yes, in what week of the pregnancy?	☐1 0-4 weeks<br>☐2 5-8 weeks<br>☐3 9-12 weeks<br>☐4 13+ weeks
(d). Have you taken any medication for this problem	☐1 Yes<br>☐2 No **[If no, skip to Q118]**
(i). Name of medication	__________
(ii). Do you have the bottle or package that you could show?<br>[Interview to indicate whether bottle or package seen:<br>Seen: Yes _____/No _____]	☐1 Yes<br>☐2 No
(iii). Was this prescribed by:	☐1 Doctor<br>☐2 Clinic nurse

	☐3 None of the above<br>☐4 Don't know
(iv). Was this over the counter medication	☐1 Yes<br>☐2 No
(v). No. of days taken	__________ days
118. Have you ever been told by a doctor that you have Sciatica	☐1 Yes<br>☐2 No **[If no, skip to Q119]**
(a). Were you told this more than six months before this pregnancy?	☐1 Yes<br>☐2 No
(b). Were you told this in the last six months before this pregnancy?	☐1 Yes<br>☐2 No
(c). Were you told this during this pregnancy?	☐1 Yes<br>☐2 No
(i). If yes, in what week of the pregnancy?	☐1 0-4 weeks<br>☐2 5-8 weeks<br>☐3 9-12 weeks<br>☐4 13+ weeks
(d). Have you taken any medication for this problem	☐1 Yes<br>☐2 No **[If no, skip to Q119]**
(i). Name of medication	__________
(ii). Do you have the bottle or package that you could show?<br>[Interview to indicate whether bottle or package seen:<br>Seen: Yes _____/No _____]	☐1 Yes<br>☐2 No
(iii). Was this prescribed by:	☐1 Doctor<br>☐2 Clinic nurse<br>☐3 None of the above<br>☐4 Don't know
(iv). Was this over the counter medication	☐1 Yes<br>☐2 No
(v). No. of days taken	__________ days
119. Have you ever been told by a doctor that you have myalgia	☐1 Yes<br>☐2 No **[If no, skip to Q120]**
(a). Were you told this more than six months before this pregnancy?	☐1 Yes<br>☐2 No
(b). Were you told this in the last six months before this pregnancy?	☐1 Yes<br>☐2 No
(c). Were you told this during this pregnancy?	☐1 Yes<br>☐2 No
(i). If yes, in what week of the pregnancy?	☐1 0-4 weeks<br>☐2 5-8 weeks<br>☐3 9-12 weeks<br>☐4 13+ weeks
(d). Have you taken any medication for this problem	☐1 Yes<br>☐2 No **[If no, skip to Q120]**
(i). Name of medication	__________
(ii). Do you have the bottle or package that you could show?<br>[Interview to indicate whether bottle or package seen:<br>Seen: Yes _____/No _____]	☐1 Yes<br>☐2 No
(iii). Was this prescribed by:	☐1 Doctor<br>☐2 Clinic nurse

	☐3 None of the above<br>☐4 Don't know
(iv). Was this over the counter medication	☐1 Yes<br>☐2 No
(v). No. of days taken	__________ days
**Genital and urinary tract**	
120. Have you ever been told by a doctor that you have Ovary/fallopian tube infection	☐1 Yes<br>☐2 No **[If no, skip to Q121]**
(a). Were you told this more than six months before this pregnancy?	☐1 Yes<br>☐2 No
(b). Were you told this in the last six months before this pregnancy?	☐1 Yes<br>☐2 No
(c). Were you told this during this pregnancy?	☐1 Yes<br>☐2 No
(i). If yes, in what week of the pregnancy?	☐1 0-4 weeks<br>☐2 5-8 weeks<br>☐3 9-12 weeks<br>☐4 13+ weeks
(d). Have you taken any medication for this problem	☐1 Yes<br>☐2 No **[If no, skip to Q121]**
(i). Name of medication	__________
(ii). Do you have the bottle or package that you could show?<br>[Interview to indicate whether bottle or package seen:<br>Seen: Yes _____/No _____]	☐1 Yes<br>☐2 No
(iii). Was this prescribed by:	☐1 Doctor<br>☐2 Clinic nurse<br>☐3 None of the above<br>☐4 Don't know
(iv). Was this over the counter medication	☐1 Yes<br>☐2 No
(v). No. of days taken	__________ days
121. Have you ever been told by a doctor that you have Endometriosis	☐1 Yes<br>☐2 No **[If no, skip to Q122]**
(a). Were you told this more than six months before this pregnancy?	☐1 Yes<br>☐2 No
(b). Were you told this in the last six months before this pregnancy?	☐1 Yes<br>☐2 No
(c). Were you told this during this pregnancy?	☐1 Yes<br>☐2 No
(i). If yes, in what week of the pregnancy?	☐1 0-4 weeks<br>☐2 5-8 weeks<br>☐3 9-12 weeks<br>☐4 13+ weeks
(d). Have you taken any medication for this problem	☐1 Yes<br>☐2 No **[If no, skip to Q122]**
(i). Name of medication	__________
(ii). Do you have the bottle or package that you could show?<br>[Interview to indicate whether bottle or package seen:<br>Seen: Yes _____/No _____]	☐1 Yes<br>☐2 No
(iii). Was this prescribed by:	☐1 Doctor<br>☐2 Clinic nurse

	☐3 None of the above<br>☐4 Don't know
(iv). Was this over the counter medication	☐1 Yes<br>☐2 No
(v). No. of days taken	__________ days
122. Have you ever been told by a doctor that you have Ovarian cyst/	☐1 Yes<br>☐2 No **[If no, skip to Q123]**
(a). Were you told this more than six months before this pregnancy?	☐1 Yes<br>☐2 No
(b). Were you told this in the last six months before this pregnancy?	☐1 Yes<br>☐2 No
(c). Were you told this during this pregnancy?	☐1 Yes<br>☐2 No
(i). If yes, in what week of the pregnancy?	☐1 0-4 weeks<br>☐2 5-8 weeks<br>☐3 9-12 weeks<br>☐4 13+ weeks
(d). Have you taken any medication for this problem	☐1 Yes<br>☐2 No **[If no, skip to Q123]**
(i). Name of medication	__________
(ii). Do you have the bottle or package that you could show?<br>[Interview to indicate whether bottle or package seen:<br>Seen: Yes _____/No _____]	☐1 Yes<br>☐2 No
(iii). Was this prescribed by:	☐1 Doctor<br>☐2 Clinic nurse<br>☐3 None of the above<br>☐4 Don't know
(iv). Was this over the counter medication	☐1 Yes<br>☐2 No
(v). No. of days taken	__________ days
123. Have you ever been told by a doctor that you have any cervical disease	☐1 Yes<br>☐2 No **[If no, skip to Q124]**
(a). Were you told this more than six months before this pregnancy?	☐1 Yes<br>☐2 No
(b). Were you told this in the last six months before this pregnancy?	☐1 Yes<br>☐2 No
(c). Were you told this during this pregnancy?	☐1 Yes<br>☐2 No
(i). If yes, in what week of the pregnancy?	☐1 0-4 weeks<br>☐2 5-8 weeks<br>☐3 9-12 weeks<br>☐4 13+ weeks
(d). Have you taken any medication for this problem	☐1 Yes<br>☐2 No **[If no, skip to Q124]**
(i). Name of medication	__________
(ii). Do you have the bottle or package that you could show?<br>[Interview to indicate whether bottle or package seen:<br>Seen: Yes _____/No _____]	☐1 Yes<br>☐2 No
(iii). Was this prescribed by:	☐1 Doctor<br>☐2 Clinic nurse

	☐3 None of the above<br>☐4 Don't know
(iv). Was this over the counter medication	☐1 Yes<br>☐2 No
(v). No. of days taken	__________ days
124. Have you ever been told by a doctor that you have sexually transmitted infections?	☐1 Yes<br>☐2 No **[If no, skip to Q125]**
(a). Were you told this more than six months before this pregnancy?	☐1 Yes<br>☐2 No
(b). Were you told this in the last six months before this pregnancy?	☐1 Yes<br>☐2 No
(c). Were you told this during this pregnancy?	☐1 Yes<br>☐2 No
(i). If yes, in what week of the pregnancy?	☐1 0-4 weeks<br>☐2 5-8 weeks<br>☐3 9-12 weeks<br>☐4 13+ weeks
(d). Have you taken any medication for this problem	☐1 Yes<br>☐2 No **[If no, skip to Q125]**
(i). Name of medication	__________
(ii). Do you have the bottle or package that you could show?<br>[Interview to indicate whether bottle or package seen:<br>Seen: Yes _____/No _____]	☐1 Yes<br>☐2 No
(iii). Was this prescribed by:	☐1 Doctor<br>☐2 Clinic nurse<br>☐3 None of the above<br>☐4 Don't know
(iv). Was this over the counter medication	☐1 Yes<br>☐2 No
(v). No. of days taken	__________ days
(e). What was this disease?	☐1 herpes<br>☐2 chlamydia<br>☐3 gonorrhoea<br>☐4 syphyllis<br>☐5 venereal warts<br>☐88 other, please describe: ___________<br>☐99 don't know
125. Have you ever been told by a doctor that you have kidney stones	☐1 Yes<br>☐2 No **[If no, skip to Q126]**
(a). Were you told this more than six months before this pregnancy?	☐1 Yes<br>☐2 No
(b). Were you told this in the last six months before this pregnancy?	☐1 Yes<br>☐2 No
(c). Were you told this during this pregnancy?	☐1 Yes<br>☐2 No
(i). If yes, in what week of the pregnancy?	☐1 0-4 weeks<br>☐2 5-8 weeks<br>☐3 9-12 weeks<br>☐4 13+ weeks
(d). Have you taken any medication for this problem	☐1 Yes<br>☐2 No **[If no, skip to Q126]**
(i). Name of medication	__________

(ii). Do you have the bottle or package that you could show?<br>[Interview to indicate whether bottle or package seen:<br>Seen: Yes _____/No _____]	☐1 Yes<br>☐2 No
(iii). Was this prescribed by:	☐1 Doctor<br>☐2 Clinic nurse<br>☐3 None of the above<br>☐4 Don't know
(iv). Was this over the counter medication	☐1 Yes<br>☐2 No
(v). No. of days taken	__________ days
126. Have you ever been told by a doctor that you have kidney infection	☐1 Yes<br>☐2 No **[If no, skip to Q127]**
(a). Were you told this more than six months before this pregnancy?	☐1 Yes<br>☐2 No
(b). Were you told this in the last six months before this pregnancy?	☐1 Yes<br>☐2 No
(c). Were you told this during this pregnancy?	☐1 Yes<br>☐2 No
(i). If yes, in what week of the pregnancy?	☐1 0-4 weeks<br>☐2 5-8 weeks<br>☐3 9-12 weeks<br>☐4 13+ weeks
(d). Have you taken any medication for this problem	☐1 Yes<br>☐2 No **[If no, skip to Q127]**
(i). Name of medication	__________
(ii). Do you have the bottle or package that you could show?<br>[Interview to indicate whether bottle or package seen:<br>Seen: Yes _____/No _____]	☐1 Yes<br>☐2 No
(iii). Was this prescribed by:	☐1 Doctor<br>☐2 Clinic nurse<br>☐3 None of the above<br>☐4 Don't know
(iv). Was this over the counter medication	☐1 Yes<br>☐2 No
(v). No. of days taken	__________ days
127. Have you ever been told by a doctor that you have urinary tract infection?	☐1 Yes<br>☐2 No **[If no, skip to Q128]**
(a). Were you told this more than six months before this pregnancy?	☐1 Yes<br>☐2 No
(b). Were you told this in the last six months before this pregnancy?	☐1 Yes<br>☐2 No
(c). Were you told this during this pregnancy?	☐1 Yes<br>☐2 No
(i). If yes, in what week of the pregnancy?	☐1 0-4 weeks<br>☐2 5-8 weeks<br>☐3 9-12 weeks<br>☐4 13+ weeks
(d). Have you taken any medication for this problem	☐1 Yes<br>☐2 No **[If no, skip to Q128]**
(i). Name of medication	__________

(ii). Do you have the bottle or package that you could show?<br>[Interview to indicate whether bottle or package seen:<br>Seen: Yes _____/No _____]	☐1 Yes<br>☐2 No
(iii). Was this prescribed by:	☐1 Doctor<br>☐2 Clinic nurse<br>☐3 None of the above<br>☐4 Don't know
(iv). Was this over the counter medication	☐1 Yes<br>☐2 No
(v). No. of days taken	__________ days
128. Have you ever been told by a doctor that you have incontinence?	☐1 Yes<br>☐2 No **[If no, skip to Q129]**
(a). Were you told this more than six months before this pregnancy?	☐1 Yes<br>☐2 No
(b). Were you told this in the last six months before this pregnancy?	☐1 Yes<br>☐2 No
(c). Were you told this during this pregnancy?	☐1 Yes<br>☐2 No
(i). If yes, in what week of the pregnancy?	☐1 0-4 weeks<br>☐2 5-8 weeks<br>☐3 9-12 weeks<br>☐4 13+ weeks
(d). Have you taken any medication for this problem	☐1 Yes<br>☐2 No **[If no, skip to Q129]**
(i). Name of medication	__________
(ii). Do you have the bottle or package that you could show?<br>[Interview to indicate whether bottle or package seen:<br>Seen: Yes _____/No _____]	☐1 Yes<br>☐2 No
(iii). Was this prescribed by:	☐1 Doctor<br>☐2 Clinic nurse<br>☐3 None of the above<br>☐4 Don't know
(iv). Was this over the counter medication	☐1 Yes<br>☐2 No
(v). No. of days taken	__________ days
**Other illnesses/health problems**	
129. Have you ever been told by a doctor that you have anorexia, bulimia or other eating disorders	☐1 Yes<br>☐2 No **[If no, skip to Q130]**
(a). Were you told this more than six months before this pregnancy?	☐1 Yes<br>☐2 No
(b). Were you told this in the last six months before this pregnancy?	☐1 Yes<br>☐2 No
(c). Were you told this during this pregnancy?	☐1 Yes<br>☐2 No
(i). If yes, in what week of the pregnancy?	☐1 0-4 weeks<br>☐2 5-8 weeks<br>☐3 9-12 weeks<br>☐4 13+ weeks
(d). Have you taken any medication for this problem	☐1 Yes<br>☐2 No **[If no, skip to Q130]**
(i). Name of medication	__________

(ii). Do you have the bottle or package that you could show?<br>[Interview to indicate whether bottle or package seen:<br>Seen: Yes _____/No _____]	☐1 Yes<br>☐2 No
(iii). Was this prescribed by:	☐1 Doctor<br>☐2 Clinic nurse<br>☐3 None of the above<br>☐4 Don't know
(iv). Was this over the counter medication	☐1 Yes<br>☐2 No
(v). No. of days taken	__________ days
130. Have you ever been told by a doctor that you have Migraine	☐1 Yes<br>☐2 No **[If no, skip to Q131]**
(a). Were you told this more than six months before this pregnancy?	☐1 Yes<br>☐2 No
(b). Were you told this in the last six months before this pregnancy?	☐1 Yes<br>☐2 No
(c). Were you told this during this pregnancy?	☐1 Yes<br>☐2 No
(i). If yes, in what week of the pregnancy?	☐1 0-4 weeks<br>☐2 5-8 weeks<br>☐3 9-12 weeks<br>☐4 13+ weeks
(d). Have you taken any medication for this problem	☐1 Yes<br>☐2 No **[If no, skip to Q131]**
(i). Name of medication	__________
(ii). Do you have the bottle or package that you could show?<br>[Interview to indicate whether bottle or package seen:<br>Seen: Yes _____/No _____]	☐1 Yes<br>☐2 No
(iii). Was this prescribed by:	☐1 Doctor<br>☐2 Clinic nurse<br>☐3 None of the above<br>☐4 Don't know
(iv). Was this over the counter medication	☐1 Yes<br>☐2 No
(v). No. of days taken	__________ days
131. Have you ever been told by a doctor that you have Epilepsy	☐1 Yes<br>☐2 No **[If no, skip to Q132]**
(a). Were you told this more than six months before this pregnancy?	☐1 Yes<br>☐2 No
(b). Were you told this in the last six months before this pregnancy?	☐1 Yes<br>☐2 No
(c). Were you told this during this pregnancy?	☐1 Yes<br>☐2 No
(i). If yes, in what week of the pregnancy?	☐1 0-4 weeks<br>☐2 5-8 weeks<br>☐3 9-12 weeks<br>☐4 13+ weeks
(d). Have you taken any medication for this problem	☐1 Yes<br>☐2 No **[If no, skip to Q132]**
(i). Name of medication	__________

(ii). Do you have the bottle or package that you could show?<br>[Interview to indicate whether bottle or package seen:<br>Seen: Yes _____/No _____]	☐1 Yes<br>☐2 No
(iii). Was this prescribed by:	☐1 Doctor<br>☐2 Clinic nurse<br>☐3 None of the above<br>☐4 Don't know
(iv). Was this over the counter medication	☐1 Yes<br>☐2 No
(v). No. of days taken	__________ days
132. Have you ever been told by a doctor that you have Cancer	☐1 Yes<br>☐2 No **[If no, skip to Q133]**
(a). Were you told this more than six months before this pregnancy?	☐1 Yes<br>☐2 No
(b). Were you told this in the last six months before this pregnancy?	☐1 Yes<br>☐2 No
(c). Were you told this during this pregnancy?	☐1 Yes<br>☐2 No
(i). If yes, in what week of the pregnancy?	☐1 0-4 weeks<br>☐2 5-8 weeks<br>☐3 9-12 weeks<br>☐4 13+ weeks
(d). Have you taken any medication for this problem	☐1 Yes<br>☐2 No **[If no, skip to Q133]**
(i). Name of medication	__________
(ii). Do you have the bottle or package that you could show?<br>[Interview to indicate whether bottle or package seen:<br>Seen: Yes _____/No _____]	☐1 Yes<br>☐2 No
(iii). Was this prescribed by:	☐1 Doctor<br>☐2 Clinic nurse<br>☐3 None of the above<br>☐4 Don't know
(iv). Was this over the counter medication	☐1 Yes<br>☐2 No
(v). No. of days taken	__________ days
133. Have you ever been told by a doctor that you have Depression or Anxiety	☐1 Yes<br>☐2 No **[If no, skip to Q134]**
(a). Were you told this more than six months before this pregnancy?	☐1 Yes<br>☐2 No
(b). Were you told this in the last six months before this pregnancy?	☐1 Yes<br>☐2 No
(c). Were you told this during this pregnancy?	☐1 Yes<br>☐2 No
(i). If yes, in what week of the pregnancy?	☐1 0-4 weeks<br>☐2 5-8 weeks<br>☐3 9-12 weeks<br>☐4 13+ weeks
(d). Have you taken any medication for this problem	☐1 Yes<br>☐2 No **[If no, skip to Q134]**
(i). Name of medication	__________

(ii). Do you have the bottle or package that you could show?<br>[Interview to indicate whether bottle or package seen:<br>Seen: Yes _____/No _____]	☐1 Yes<br>☐2 No
(iii). Was this prescribed by:	☐1 Doctor<br>☐2 Clinic nurse<br>☐3 None of the above<br>☐4 Don't know
(iv). Was this over the counter medication	☐1 Yes<br>☐2 No
(v). No. of days taken	_________ days
134. Have you ever been told by a doctor that you have any other long illnesses or health problems	☐1 Yes<br>☐2 No **[If no, skip to Q135]**
If so, please describe	___________________
(a). Were you told this more than six months before this pregnancy?	☐1 Yes<br>☐2 No
(b). Were you told this in the last six months before this pregnancy?	☐1 Yes<br>☐2 No
(c). Were you told this during this pregnancy?	☐1 Yes<br>☐2 No
(i). If yes, in what week of the pregnancy?	☐1 0-4 weeks<br>☐2 5-8 weeks<br>☐3 9-12 weeks<br>☐4 13+ weeks
(d). Have you taken any medication for this problem	☐1 Yes<br>☐2 No **[If no, skip to Q135]**
(i). Name of medication	_________
(ii). Do you have the bottle or package that you could show?<br>[Interview to indicate whether bottle or package seen:<br>Seen: Yes _____/No _____]	☐1 Yes<br>☐2 No
(iii). Was this prescribed by:	☐1 Doctor<br>☐2 Clinic nurse<br>☐3 None of the above<br>☐4 Don't know
(iv). Was this over the counter medication	☐1 Yes<br>☐2 No
(v). No. of days taken	_________ days
**Other diseases**	
135. Do you have a congenital deformity or birth defect?	☐1 Yes<br>☐2 No
136. If yes, please describe?	__________________________
137. Do your gums bleed when you brush your teeth at present?	☐1 No, rarely or never<br>☐2 Yes, sometimes<br>☐3 Yes, often<br>☐4 Yes, almost always
**H. OTHER MEDICATION**	
138. Have you used other medication not previously mentioned?	☐1 Yes<br>☐2 No **[If no, skip to Q145]**
139. If yes, please state these medication	Medication 1:<br>________________________

	Medication 2: ________________________<br>Medication 3: ________________________<br>Medication 4: ________________________<br>Medication 5: ________________________
140. Do you have the bottle or package that you could show for medication 1?<br>[Interview to indicate whether bottle or package seen: Seen: Yes _____/No _____]	☐1 Yes<br>☐2 No
(a). Was this prescribed by:	☐1 Doctor<br>☐2 Clinic nurse<br>☐3 None of the above<br>☐4 Don't know
(b). Was this over the counter medication	☐1 Yes<br>☐2 No
(c). No. of days taken	_________ days
(d). Did you take this more than six months before this pregnancy?	☐1 Yes<br>☐2 No
(e). Did you take this during this pregnancy?	☐1 Yes<br>☐2 No
(f). If yes, in what week of the pregnancy?	☐1 0-4 weeks<br>☐2 5-8 weeks<br>☐3 9-12 weeks<br>☐4 13+ weeks
141. Do you have the bottle or package that you could show for medication 2?<br>[Interview to indicate whether bottle or package seen: Seen: Yes _____/No _____]	☐1 Yes<br>☐2 No
(a). Was this prescribed by:	☐1 Doctor<br>☐2 Clinic nurse<br>☐3 None of the above<br>☐4 Don't know
(b). Was this over the counter medication	☐1 Yes<br>☐2 No
(c). No. of days taken	_________ days
(d). Did you take this more than six months before this pregnancy?	☐1 Yes<br>☐2 No
(e). Did you take this during this pregnancy?	☐1 Yes<br>☐2 No
(f). If yes, in what week of the pregnancy?	☐1 0-4 weeks<br>☐2 5-8 weeks<br>☐3 9-12 weeks<br>☐4 13+ weeks
142. Do you have the bottle or package that you could show for medication 3?<br>[Interview to indicate whether bottle or package seen: Seen: Yes _____/No _____]	☐1 Yes<br>☐2 No
(a). Was this prescribed by:	☐1 Doctor<br>☐2 Clinic nurse<br>☐3 None of the above<br>☐4 Don't know

(b). Was this over the counter medication	☐1 Yes<br>☐2 No
(c). No. of days taken	_________ days
(d). Did you take this more than six months before this pregnancy?	☐1 Yes<br>☐2 No
(e). Did you take this during this pregnancy?	☐1 Yes<br>☐2 No
(f). If yes, in what week of the pregnancy?	☐1 0-4 weeks<br>☐2 5-8 weeks<br>☐3 9-12 weeks<br>☐4 13+ weeks
143. Do you have the bottle or package that you could show for medication 4?<br>[Interview to indicate whether bottle or package seen:<br>Seen: Yes _____/No _____]	☐1 Yes<br>☐2 No
(a). Was this prescribed by:	☐1 Doctor<br>☐2 Clinic nurse<br>☐3 None of the above<br>☐4 Don't know
(b). Was this over the counter medication	☐1 Yes<br>☐2 No
(c). No. of days taken	_________ days
(d). Did you take this more than six months before this pregnancy?	☐1 Yes<br>☐2 No
(e). Did you take this during this pregnancy?	☐1 Yes<br>☐2 No
(f). If yes, in what week of the pregnancy?	☐1 0-4 weeks<br>☐2 5-8 weeks<br>☐3 9-12 weeks<br>☐4 13+ weeks
144. Do you have the bottle or package that you could show for medication 5?<br>[Interview to indicate whether bottle or package seen:<br>Seen: Yes _____/No _____]	☐1 Yes<br>☐2 No
(a). Was this prescribed by:	☐1 Doctor<br>☐2 Clinic nurse<br>☐3 None of the above<br>☐4 Don't know
(b). Was this over the counter medication	☐1 Yes<br>☐2 No
(c). No. of days taken	_________ days
(d). Did you take this more than six months before this pregnancy?	☐1 Yes<br>☐2 No
(e). Did you take this during this pregnancy?	☐1 Yes<br>☐2 No
(f). If yes, in what week of the pregnancy?	☐1 0-4 weeks<br>☐2 5-8 weeks<br>☐3 9-12 weeks<br>☐4 13+ weeks
**I WORK AND LEISURE**	
145. What was your work situation when you became pregnant? (*Fill on one or several boxes for each.)*	☐1 Student [skip to Q162]<br>☐2 At home [skip to Q162]<br>☐3 Intern/apprentice<br>☐4 Military service

	☐$_5$ Unemployed/laid off<br>☐$_6$ Rehabilitation/disabled [skip to Q162]<br>☐$_7$ Employed in public sector<br>☐$_8$ Self-employed<br>☐$_9$ Family member without steady income in family company<br>☐$_{10}$ manufacturing<br>☐$_{11}$ chemical<br>☐$_{12}$ mining<br>☐$_{13}$ commercial and retail<br>☐$_{14}$ agricultural and farming<br>☐$_{88}$ Other ____________________________
146. What kind of work do/did you do?	☐$_1$ general assistant<br>☐$_2$ clerical/administrative<br>☐$_3$ machine operator<br>☐$_4$ farm assistant<br>☐$_5$ supervisor/manager<br>☐$_6$ engineering, designer or planning<br>☐$_{88}$ other, please specify: ____________
147. Did you have an extra job (with or without salary) when you became pregnant? *(For example, accountant, hair dresser, singer in a dance band, club leader)*	☐$_1$ Yes<br>☐$_2$ No<br>Describe: ______________________
148. Have you been absent from work more than two weeks during this pregnancy?	☐$_1$ Yes<br>☐$_2$ No
149. Are you absent from your work at the present time?	☐$_1$ Yes<br>☐$_2$ No
150. If yes, what is the reason for your absence? *(Fill in one or several boxes.)*	☐$_1$ Medical leave<br>☐$_2$ Leave of absence<br>☐$_3$ Sick child<br>☐$_{88}$ Other: _______________________________
151 The usual number of paid working hours a week before you became pregnant and at present.	Before the pregnancy hours_________________<br>During the pregnancy hours ________________
152. Describe the type of work carried out at your place of work as accurately as possible. (*Write for example, hospital department for children with cancer, body shop at a garage for diesel vehicles, farming with grain and swine, work in the home.)*	________________________________<br>________________________________<br>________________________________<br>________________________________<br>________________________________<br>________________________________<br>________________________________<br>_______________________

Indicate the appropriate answer for each of the following questions concerning your present work situation, using the following score:
1 = yes, everyday, more than half of the working day
2 = yes, everyday, less than half of the working day
3 = yes, periodically, but not daily
4 = seldom or never
(Fill in only one box in each line.)

If the respondent is not involved in any occupational activity, then **skip to Q162.** For the purposes of these questions, housework is included as occupational activity				
153. Too much to do, that the work situation is taxing	1	2	3	4
154. Bending and turning many times in an hour				
155. Working with hands above shoulder level				
156. Working in a standing or walking position				
157. Can choose to work faster on some days, and slower on some days				
158. Have a lot of uncomfortable background noise				
159. Have a lot of background noise that causes you to raise your voice when talking to others even within a distance of one metre				
160. When are your working hours? *(Fill in one or several boxes.)*	☐1 Permanent day work<br>☐2 Permanent afternoon or evening work<br>☐3 Permanent night work<br>☐4 Shift work (day and night) or shift rotations<br>☐5 No set times (extra work, extra shifts, temporary employment, etc.)/<br>☐88 Other<br>___________________________________			
161. During your pregnancy do you lift anything that weighs more than 10 kg at work? (*10 kilos is the equivalent of a full bucket of water.)*	☐1 .Infrequently or never<br>☐2 Yes, less than 20 times a week<br>☐3 Yes, more than 20 times a week<br>☐4 Yes, 10-20 times a day<br>☐5 Yes, more than 20 times a day			
162. During your pregnancy do you lift anything that weighs more than 10 kg at home? (*10 kilos is the equivalent of a full bucket of water.)*	☐1 Infrequently or never<br>☐2 Yes, less than 20 times a week<br>☐3 Yes, more than 20 times a week<br>☐4 Yes, 10-20 times a day<br>☐5 Yes, more than 20 times a day			
163. What was the baby's father work situation when you became pregnant? (*Fill on one or several boxes for each.)*	☐1 Student [skip to Q166]<br>☐2 At home [skip to Q166]<br>☐3 Intern/apprentice<br>☐4 Military service<br>☐5 Unemployed/laid off<br>☐6 Rehabilitation/disabled [skip to Q166]<br>☐7 Employed in public sector<br>☐8 Self-employed<br>☐9 Family member without steady income in family company<br>☐10 manufacturing<br>☐11 chemical<br>☐12 mining<br>☐13 commercial and retail<br>☐14 agricultural and farming<br>☐88 Other<br>______________________________			
164. Describe the type of work carried out at the baby's father's place of work as accurately as possible. (*Write for example, hospital department for children*	___________________________________<br>___________________________________<br>___________________________________			

with cancer, body shop at a garage for diesel vehicles, farming with grain and swine, work in the home.)	________________________________<br>________________________________<br>__________________________
165. How often do you use a cell phone?	☐1 Infrequently<br>☐2 Never<br>☐3 A few times a week<br>☐4 Daily<br>☐5 On average more than an hour daily
166. How often do you work with a computer, laser printer or copying machine (at a distance of less than two metres) after you became pregnant?	☐1 Never<br>☐2 A few times a week<br>☐3 Daily<br>☐4 On average more than an hour daily
167. How often have you worked with x-ray equipment (at a distance of less than two metres) after you became pregnant? (*This does not include treatment as a patient*)	☐1 Never<br>☐2 A few times a week<br>☐3 Daily<br>☐4 On average more than an hour daily
**Have you been in contact with any of the following substances either at work or in your leisure time during the last six months, and if yes, for how many days?**	
168. Lead vapors, lead dust, lead particles or lead alloys	☐1 Yes<br>☐2 No
(a). If yes, for how many days (daily = 180 days)	___________ days
169. Chrome, arsenic, cadmium or combinations of these	☐1 Yes<br>☐2 No
(a). If yes, for how many days (daily = 180 days)	___________ days
170. petrol or exhaust vapour (does not apply to filling gasoline in your own car)	☐1 Yes<br>☐2 No
(a). If yes, for how many days (daily = 180 days)	___________ days
171. Mercury vapors, mercury or work with amalgam fillings (does not apply to your own dental treatment)	☐1 Yes<br>☐2 No
(a). If yes, for how many days (daily = 180 days)	___________ days
172. Disinfectants, vermin poisons	☐1 Yes<br>☐2 No
(a). If yes, for how many days (daily = 180 days)	___________ days
173. Weed killers, insecticides, fungicides	☐1 Yes<br>☐2 No
(a). If yes, for how many days (daily = 180 days)	___________ days
174. Oil-based paint	☐1 Yes<br>☐2 No
(a). If yes, for how many days (daily = 180 days)	___________ days
175. Substances used in welding	☐1 Yes<br>☐2 No
(a). If yes, for how many days (daily = 180 days)	___________ days
176. Substances used in soldering	☐1 Yes<br>☐2 No
(a). If yes, for how many days (daily = 180 days)	___________ days
177. Formalin/ formaldehyde	☐1 Yes<br>☐2 No
(a). If yes, for how many days (daily = 180 days)	___________ days
178. Photographic chemicals (fixatives or developers)	☐1 Yes<br>☐2 No
(a). If yes, for how many days (daily = 180 days)	___________ days
179. Motor oil, lubrication oil or other types of oil	☐1 Yes<br>☐2 No

(a). If yes, for how many days (daily = 180 days)	___________ days
180. Industrial dyes or ink	☐$_1$ Yes<br>☐$_2$ No
(a). If yes, for how many days (daily = 180 days)	___________ days
181. Chemotherapeutic substances/chemotherapy treatment (does not apply to your own medical treatment)	☐$_1$ Yes<br>☐$_2$ No
(a). If yes, for how many days (daily = 180 days)	___________ days
182. Laughing gas or other anesthetic gases (does not apply to you own treatment as a patient)	☐$_1$ Yes<br>☐$_2$ No
(a). If yes, for how many days (daily = 180 days)	___________ days
183. Water-based or latex paint	☐$_1$ Yes<br>☐$_2$ No
(a). If yes, for how many days (daily = 180 days)	___________ days
184. Paint thinner, paint-lacquer-glue remover or other solvents (ex. lynol, turpentine, toluene, carbon tetrachloride)	☐$_1$ Yes<br>☐$_2$ No
(a). If yes, for how many days (daily = 180 days)	___________ days
185. Other substances and conditions, describe:_____	☐$_1$ Yes<br>☐$_2$ No
(a). If yes, for how many days (daily = 180 days)	___________ days
186. How often have you been to a night club since you became pregnant?	☐$_1$ 1-2 times a week<br>☐$_2$ Less often<br>☐$_3$ Never
187. Are you in contact with animals either at work or in your leisure?	☐$_1$ Yes<br>☐$_2$ No **[If no, skip to Q202]**

If yes, what sort of animals?
Please indicate how often are you in contact with them on a weekly basis, using the following code:
0 = never
1 = daily
2 = 3-6 times a week
3 = 1-2 times a week
4 = less than once a week

	0	1	2	3	4	
188. Dog						
189. Cat						
190. Guinea Pig						
191. Hamster						
192. Rabbit						
193. Bird						
194. Aquarium Fish						
195. Cow						
196 Pig						
197 Sheep, goat						
199. Horse						
200. Poultry						
201. Other: ____________						

**J. HABITS**		
202. Did your mother smoke when she was pregnant with you?	☐$_1$ Yes<br>☐$_2$ No<br>Don't know	
203. Are you exposed to passive smoking at home?	☐$_1$ Yes	

	☐2 No	
204. If yes, how many hours a day are you exposed to passive smoking?	_______________ hours a day	
2056. Are you exposed to passive smoking at work?	☐1 Yes<br>☐2 No	
206. If yes, how many hours a day are you exposed to passive smoking?	_______________hours a day	
207. Did the baby's father smoke before you became pregnant?	☐1 Yes<br>☐2 No	
208. Does he smoke now?	☐1 Yes<br>☐2 No	
209. Have you ever smoked?	☐1 Yes<br>☐2 No (proceed question 104)	
210. Do you smoke now (after you became pregnant)?	☐1 No<br>☐2 Sometimes<br>☐3 Daily	
(a). If yes, how many	__________ cigarettes per week<br>__________ cigarettes per day	
211. Did you smoke during the last 3 months before you became pregnant this time?	☐1 No<br>☐2 Sometimes<br>☐3 Daily	
(a). If yes, how many	__________ cigarettes per week<br>__________ cigarettes per day	
212. How old were you when you started to smoke on a daily basis?	___________ years	
213. Have you stopped smoking completely?	☐1 Yes<br>☐2 No	
214. If yes, how old were you when you stopped smoking?	_____________ years	
215. If you stopped smoking after you became pregnant, in which week of pregnancy did you stop?	_____________ week of pregnancy	

Have you used any of the following substances?
If yes, please indicate when you used them, using the following code:
1 = never
2 = previously
3 = last month before this pregnancy
4 = during this pregnancy

216. Hash	1	2	3	4	
217. Ecstasy					
218. Heroin					
219. Cocaine					
220. Amphetamine					
221. Other: ________					

222. Have you ever consumed alcohol?	☐1 Yes<br>☐2 No **[If no, skip to Q230]**	

Alcohol units are used to compare the different types of alcoholic beverages. 1 alcohol unit (= 1.5 cl. pure alcohol) is equivalent to:
1 bottle/can energy drink or cider
1 glass (1/3 liter) of beer
1 wine glass red or white wine
1 wine glass sherry or other fortified wine

1 snaps glass spirits or liqueur		
223. How often did you consume alcohol in the 3 months before you became pregnant?	☐1 Approximately 6-7 time a week<br>☐2 Approximately 4-5 times a week<br>☐3 approximately 2-3 times a week<br>☐4 approximately once a week<br>☐5 approximately 1-3 times a month<br>☐6 less than once a month<br>☐7 Never	
224. How often do you consume alcohol during this pregnancy?	☐1 Approximately 6-7 time a week<br>☐2 Approximately 4-5 times a week<br>☐3 approximately 2-3 times a week<br>☐4 approximately once a week<br>☐5 approximately 1-3 times a month<br>☐6 less than once a month<br>☐7 Never	
225. What type of alcohol do you usually drink? *(Fill in one or several boxes.)*	☐1 Light beer<br>☐2 Beer<br>☐3 Red wine<br>☐4 White wine<br>☐5 Low alcohol sodas<br>☐6 Fortified wines (*sherry, port wine, Madeira)*/ ☐7 Spirits (*vodka, gin, snaps, cognac, whisky, liqueur)*	
226. Did you drink 5 units or more at least once during the last 3 months before this pregnancy?	☐1 Several times per week<br>☐2 Once a week<br>☐3 1-3 times a month<br>☐4 Less than once a month<br>☐5 Never	
227. Did you drink 5 units or more at least once during this pregnancy?	☐1 Several times per week<br>☐2 Once a week<br>☐3 1-3 times a month<br>☐4 Less than once a month<br>☐5 Never	
228. How many units of alcohol did you usually drink when you consume alcohol in the last 3 months before pregnancy?	☐1 10 or more<br>☐2 7-9<br>☐3 5-6<br>☐4 3-4<br>☐4 1-2<br>☐5 Less that 1	
229. How many units of alcohol do you usually drink when you consume alcohol during this pregnancy?	☐1 10 or more<br>☐2 7-9<br>☐3 5-6<br>☐4 3-4<br>☐4 1-2<br>☐5 Less that 1	
**K. WEIGHT AND WEIGHT CONTROL**		
230. Do you think you were overweight before this pregnancy?	☐1 Yes, a lot<br>☐2 Yes, a little<br>☐3 No	
231. Are you worried about putting on more weight than necessary during this pregnancy?	☐1 Yes, very worried<br>☐2 Somewhat worried<br>☐3 No, not especially worried	
232. Has anyone said that you were too thin while you felt that you were too fat during the last 2 years?	☐1 Yes, often/<br>☐2 Yes, occasionally<br>☐3 No	

Have you ever used any of the following to control your weight in the last 6 months **before this pregnancy**? If yes, please state how often, using the following codes:
1 = at least once a week
2 = seldom
3 = never

233. Vomiting	1	2	3	
234. Laxatives				
235. Fasting				
236. Hard physical exercise				

Have you ever used any of the following to control your weight **during this pregnancy**? If yes, please state how often, using the following codes:
1 = at least once a week
2 = seldom
3 = never

237. Vomiting	1	2	3	
238. Laxatives				
239. Fasting				
240. Hard physical exercise				

L. PHYSICAL ACTIVITY

How often did you usually exercise in the last 3 months **before this pregnancy**? For each exercise, please indicate if you do this, and if yes, please indicate how often, using the following code:
1 = never
2 = 1-3 times per month
3 = once a week
4 = twice a week
5 = 3 times or more week
(Fill in each line, both before and during this pregnancy.)

	1	2	3	4	5	
241. Walking						
242. Brisk walking						
243. Running/jogging/cross-country running						
244. Bicycling						
245. weight training						
246. Special gymnastics/aerobics for pregnant women						
247. Aerobics/gymnastics/dance without running and jumping						
248. Aerobics/gymnastics with running and jumping						
249. Dancing						
2450. Team sports						
251. Swimming						
252. Water aerobics						
253. Riding						
254. Other						

How often do you usually exercise **during this pregnancy**?
For each exercise, please indicate if you do this, and if yes, please indicate how often, using the following code:
1 = never
2 = 1-3 times per month
3 = once a week
4 = twice a week
5 = 3 times or more week
(Fill in each line, both before and during this pregnancy.)

255. Walking	1	2	3	4	5	
256. Brisk walking						

257. Running/jogging/cross-country running						
258. Bicycling						
259. weight training						
260. Special gymnastics/aerobics for pregnant women						
261. Aerobics/gymnastics/dance without running and jumping						
262. Aerobics/gymnastics with running and jumping						
263. Dancing						
264. Team sports						
265. Swimming						
266. Water aerobics						
267. Riding						
268. Other						

269. How often were you so physically active **in your leisure time** that you get out of breath or sweat in the last 3 months before this pregnancy?	☐$_1$ Never<br>☐$_2$ Less than once a week<br>☐$_3$ Once a week<br>☐$_4$ 2 times a week<br>☐$_5$ 3-4 times a week<br>☐$_6$ 5 times a week or more	
270 How often are you currently so physically active **in your leisure time** that you get out of breath or sweat during this pregnancy?	☐$_1$ Never<br>☐$_2$ Less than once a week<br>☐$_3$ Once a week<br>☐$_4$ 2 times a week<br>☐$_5$ 3-4 times a week<br>☐$_6$ 5 times a week or more	
271. How often were you so physically active **at work** that you get out of breath or sweat in the last 3 months before this pregnancy?	☐$_1$ Never<br>☐$_2$ Less than once a week<br>☐$_3$ Once a week<br>☐$_4$ 2 times a week<br>☐$_5$ 3-4 times a week<br>☐$_6$ 5 times a week or more	
272. How often are you currently so physically active **at work** that you get out of breath or sweat during this pregnancy?	☐$_1$ Never<br>☐$_2$ Less than once a week<br>☐$_3$ Once a week<br>☐$_4$ 2 times a week<br>☐$_5$ 3-4 times a week<br>☐$_6$ 5 times a week or more	

**We would be grateful if you would write anything else you would like to tell us about this pregnancy or previous births/pregnancies that are not addressed in this questionnaire on the next page.**	
**COMMENTS**	

273. Are your contact numbers the same?	☐$_1$ Yes **[if yes, skip to Q274]**<br>☐$_2$ No	
274. If NO, what is your new contact number/s?	Home:_________________<br>Cell:___________________<br>Other:__________________	
275. Are you living at the same address?	☐$_1$ Yes **[if yes, skip to Q283]**<br>☐$_2$ No<br>☐$_{99}$ Refused to answer **[skip to Q283]**	
276. If No, What is your new address?	________________	

	House No.<br>___________________________________<br>Road/Street<br>___________________________________<br>City<br>____________________<br>Postal Code	
277. When did you move to your new address?	__________month __________year	
278. What type of new house do you currently live in?	☐1 Detached house, Semidetached<br>☐2 Farm<br>☐3 Flat, Terraced flat, Apartment building<br>☐4 Refused to answer<br>☐88 Other ____________________	
279. Has there been water damage, visible signs of fungus/mildew or a smell of mildew in your new home ? *(Fill in one or several boxes.)*	☐1 No<br>☐2 Yes, water damage<br>☐3 Yes, signs of fungus and mould<br>☐4 Yes, a smell of mildew<br>☐99 don't know	
280. Is your new home drinking water from the tap or from a river or dam?	☐1 tap<br>☐2 river or dam<br>☐3 well<br>☐88 other, please specify:<br>________________________	
281. Have you used a room heater in one or more rooms in this house?	☐1 Yes<br>☐2 No **[if No, skip to Q281]**<br>☐99 don't know **(skip to Q281)**	
(a). Was this heater fueled by	☐1 paraffin<br>☐2 gas<br>☐3 electricity<br>☐4 wood<br>☐5 coal<br>☐88 other, please specify: ______________<br>☐99 don't know	
282. Is a stove or oven used for cooking in this house?	☐1 Yes<br>☐2 No **[if No, skip to Q282]**<br>☐99 Don't know **[skip to Q282]**	
(a). What is the primary source of heat for this stove or oven?	☐1 paraffin<br>☐2 gas<br>☐3 electricity<br>☐4 wood<br>☐5 coal<br>☐88 other, please specify: ________________<br>☐99 don't know	
283. Have you been scanned by ultrasound during the pregnancy?	☐1 Yes<br>☐2 No **[if No,skip to Q286]**<br>☐99 Don't know **[skip to Q286]**	
284. If Yes, At what week of pregnancy were you scanned?	__________weeks	
285. Did the ultrasound show normal conditions?	☐1 Yes **[If yes, skip to Q286]**<br>☐2 No<br>☐99 Don't know **[skip to Q286]**	
286. If No, please explain		

287. Have you had bleedings from the vagina at anytime during this pregnancy?	☐1 Yes<br>☐2 No **[If No, skip to Q288]**<br>☐99 Don't know **[skip to Q288]**	
288. What is the reason for your bleeding?	Explain:	
289. Did you have any contractions?	☐1 Yes<br>☐2 No<br>☐99 Don't know	

Thank you!
